# Genomic and Metabolomic Analysis of the Endophytic Fungus *Fusarium* sp. VM-40 Isolated from the Medicinal Plant *Vinca minor*

**DOI:** 10.3390/jof9070704

**Published:** 2023-06-27

**Authors:** Ting He, Xiao Li, Riccardo Iacovelli, Thomas Hackl, Kristina Haslinger

**Affiliations:** 1 Department of Chemical and Pharmaceutical Biology, Groningen Research Institute of Pharmacy, University of Groningen, Antonius Deusinglaan 1, 9713 AV Groningen, The Netherlands; t.he@rug.nl (T.H.); xiao.li@rug.nl (X.L.); r.iacovelli@rug.nl (R.I.); 2 Groningen Institute of Evolutionary Life Sciences, University of Groningen, Nijenborgh 7, 9747 AG Groningen, The Netherlands; t.hackl@rug.nl

**Keywords:** endophytic fungus, *Fusarium* sp. VM-40, whole genome sequence, metabolomics, biosynthetic gene clusters, molecular networking

## Abstract

The genus *Fusarium* is well-known to comprise many pathogenic fungi that affect cereal crops worldwide, causing severe damage to agriculture and the economy. In this study, an endophytic fungus designated *Fusarium* sp. VM-40 was isolated from a healthy specimen of the traditional European medicinal plant *Vinca minor*. Our morphological characterization and phylogenetic analysis reveal that *Fusarium* sp. VM-40 is closely related to *Fusarium paeoniae,* belonging to the *F. tricinctum* species complex (FTSC), the genomic architecture and secondary metabolite profile of which have not been investigated. Thus, we sequenced the whole genome of *Fusarium* sp. VM-40 with the new Oxford Nanopore R10.4 flowcells. The assembled genome is 40 Mb in size with a GC content of 47.72%, 15 contigs (≥50,000 bp; N 50~4.3 Mb), and 13,546 protein-coding genes, 691 of which are carbohydrate-active enzyme (CAZyme)-encoding genes. We furthermore predicted a total of 56 biosynthetic gene clusters (BGCs) with antiSMASH, 25 of which showed similarity with known BGCs. In addition, we explored the potential of this fungus to produce secondary metabolites through untargeted metabolomics. Our analyses reveal that this fungus produces structurally diverse secondary metabolites of potential pharmacological relevance (alkaloids, peptides, amides, terpenoids, and quinones). We also employed an epigenetic manipulation method to activate cryptic BGCs, which led to an increased abundance of several known compounds and the identification of several putative new compounds. Taken together, this study provides systematic research on the whole genome sequence, biosynthetic potential, and metabolome of the endophytic fungus *Fusarium* sp. VM-40.

## 1. Introduction

Endophytic fungi represent an important and rich group of microorganisms that live in plant tissues or intercellular spaces and can establish beneficial relationships with host plants [1]. Many of them are promising suppliers of multiple natural products, including alkaloids, terpenoids, flavonoids, steroids, and phenolic compounds, which contribute to various interesting pharmacological effects, such as anti-inflammatory, anti-tumor, anti-phytopathogenic, antibacterial, antifungal, antiproliferative, and antioxidant activities [2,3].

*Fusarium* is one of the most common fungal genera and ubiquitously exists in terrestrial and marine environments. This genus, when associated with plants, can adopt diverse lifestyles, including saprotrophic, endophytic, and pathogenic lifestyles. Most previous studies have focused on plant pathogenicity, but more recently, scientific interest in endophytic *Fusarium* species has risen [4]. *Fusarium* endophytes have been reported to produce secondary metabolites with diverse pharmacological activities, such as paclitaxel produced by *Fusarium solani* isolated from *Taxus celebica* [5], vitexin produced by *Fusarium solani* G6 from *Cajanus cajan* [6], and quinine and cinchonidine produced by *Fusarium* isolates from *Cinchona calisaya* [7]. In addition, endophytic fungi have been investigated as promising biocontrol agents against many plant pathogens [8,9,10]. For instance, *Fusarium oxysporum* Fo47 is effective in controlling *Fusarium* wilt in tomatoes [11], and *Fusarium commune* W5 controls bakanae disease on rice flowers [12]. In light of increasing fungicide resistance and the emergence of new plant-pathogenic strains, it is a timely endeavor to further explore the antimicrobial potential of fungal endophytes and their secondary metabolites.

Fusaria are famous for their biosynthetic potential for the production of secondary metabolites (SMs), including alkaloids, peptides, amides, terpenoids, quinones, and pyranones [13]. Despite their great potential for producing diverse SMs, it is known that the majority of biosynthetic gene clusters (BGCs) remain silent under standard laboratory conditions. This indicates that a great number of novel metabolites are yet to be discovered via the activation of such silent gene clusters. Researchers have developed a variety of strategies to activate these cryptic BGCs [14,15,16]. One of these strategies involves the application of small molecular compounds that modify chromatin remodeling, ultimately leading to the induction of silent fungal BGCs [17]. Sodium butyrate (SB), which inhibits histone deacetylases, is frequently used as an inhibitor in filamentous fungi to enhance the chemical diversity of secondary metabolites [18,19].

In this study, we isolated an endophytic fungus, *Fusarium* sp. VM-40, from healthy leaves of *Vinca minor*. Our morphological identification and phylogenetic analyses of *Fusarium* sp. VM-40 indicate that this strain belongs to the *F. tricinctum* species complex, the genomic architecture and secondary metabolite profile of which have not been investigated. Herein, we explore the genome and metabolome of *Fusarium* sp. VM-40 to disclose its biosynthetic potential. In addition, we successfully employed an epigenetic manipulation strategy to increase the chemical diversity of *Fusarium* sp. VM-40. These findings provide insight into the biotechnological potential of *Fusarium* sp. VM-40.

## 2. Materials and Methods

### 2.1. Fungus Isolation and Cultivation

The *Fusarium* strain was isolated from healthy-looking, surface-sterilized leaves of *Vinca minor*. Briefly, leaves of *Vinca minor* were freshly collected in Groningen (The Netherlands) in November 2021 and washed in an ultrasonic water bath (160 W, 15 min) to remove surface dirt and adherent epiphytes. Leaves were surface-sterilized in 70% ethanol for 1 min, followed by 1% sodium hypochlorite for 2 min, then washed in distilled water for 3 × 1 min [20]. Leaves were aseptically cut into small fragments and directly placed on potato dextrose agar (PDA) medium, supplemented with 100 mg·L^−1^ ampicillin and 30 mg·L^−1^ kanamycin to prevent bacterial growth, and incubated at 28 °C for 2 to 4 weeks. A 200-microliter aliquot of water from the last washing step was also inoculated onto a PDA plate and incubated at 28 °C for the same time to check the effectiveness of surface sterilization. Fungal mycelium emerging from the leaf pieces was picked and purified by restreaking on fresh PDA medium. Plates with the purified colonies were sealed with parafilm and stored at 4 °C.

### 2.2. Morphological Analysis and Internal Transcribed Spacer (ITS)-Based Identification

For morphological characterization, the fungal isolate was grown on Synthetically nutrient-poor agar (SNA), Czapek yeast autolysate agar (CYA), and PDA media. After 7 days, the fungal colonies on each medium were observed for colony color and diameter, medium color around the colony, and colony reverse color. For microscopic analysis, samples of a small portion of the mycelium were prepared by mixing it with lactophenol blue dye and observed using an optical microscope (Olympus BX41). The digital images were captured using a connected Leica camera (Heerbrugg, Switzerland).

The full ITS region was amplified by polymerase chain reaction (PCR) with the ITSF1 (5′CTTGGTCATTTAGAGGAAGTAA3′) and ITS4 (5′TCCTCCGCTTATTGATATGC3′) primers. The PCR reaction was performed in a thermal cycler with a 2 × Q5 PCR master mix (New England Biolabs, Ipswich, MA, USA) and fungal DNA with the following program: 1 min at 98 °C, 30 cycles of 10 s at 98 °C, 15 s at 55 °C, 20 s at 72 °C, followed by 5 min of final extension at 72 °C. Two microliters of the PCR product were taken for 1% agarose gel electrophoresis analysis to confirm the successful amplification. The PCR product was purified using the QIAquick PCR Purification Kit (Qiagen, Venlo, the Netherlands) and sent to Macrogen Europe (Amsterdam, the Netherlands) for Sanger sequencing. The resulting sequences were analyzed with the Basic Local Alignment Search Tool (BLAST) against the nucleotide collection of the National Center for Biotechnology Information (NCBI) to identify the best match for the fungal isolate based on E-value.

### 2.3. Whole Genome Sequencing and Assembly

#### 2.3.1. DNA Extraction

The fungal isolate was grown at 25 °C for approximately 5 days in 25 mL Dextrose peptone yeast medium (dextrose 20 g·L^−1^, peptone 10 g·L^−1^, yeast extract 5 g·L^−1^, MgSO_4_·7H_2_O 0.5 g·L^−1^, and KH_2_PO_4_ 5 g·L^−1^), with shaking at 150 rpm. The mycelium was collected via filtration with sterilized Miracloth (Merck Millipore. Burlington, MA, USA) and washed with 20 mL of MilliQ-water, flash-frozen in liquid nitrogen, lyophilized overnight using Lyovapor™ L-200, and stored at −20 °C.

DNA extraction was performed with the Genomic Buffer Set (Qiagen) according to the manufacturer’s protocol with the following minor modifications: (1) Six 2 mL Eppendorf tubes with 25 mg lyophilized and ground mycelium were used instead of cells directly from the medium; (2) Vinotaste PRO (Novozymes, Bagsværd, Denmark) with a final concentration of 20 mg·mL^−1^ was used as lysing enzyme instead of lyticase; (3) enzymatic degradation of the cell wall was performed at 30 °C for 1 h (100 rpm) and cell lysis was performed at 50 °C for 2 h (25 rpm) instead of the recommended time and temperature. The extracted DNA was then purified using QIAGEN Genomic-Tips 20 G-1 according to the manufacturer’s protocol, with the modification that the tips were washed four times.

Circulomics Short Read Eliminator XS (PacBio, Menlo Park, CA, USA) was used to remove small fragments from the DNA preparations according to the manufacturer’s protocol. Quality control of the purified DNA was performed using NanoDrop N-100 (ThermoFisher, Waltham, MA, USA), Qubit 3.0 (Invitrogen, Waltham, MA, USA), and the Qubit dsDNA HS Assay Kit.

#### 2.3.2. Library Preparation and Sequencing

For long-read sequencing, the genomic DNA was prepared using Oxford Nanopore Technologies’ Ligation kit (SQK-LSK112) according to the manufacturer’s guidelines. Briefly, genomic DNA (1000 ng) was subjected to end repair and tailing by NEBNext FFPE DNA Repair mix and NEBNext Ultra II End repair/dA-tailing modules (New England Biolabs, Ipswich, MA, USA) and purified with AMPure XP (Beckman Coulter, Pasadena, CA, USA) magnetic beads. The sequencing adaptors were ligated using the NEBNext Quick Ligation Module (New England Biolabs, Ipswich, MA, USA). After a final product clean-up using the Long Fragment Buffer, the sequencing library was loaded into a primed FLO-MIN112 (ID: FAT75549) flow cell on a MinION device for a 46-hour run. Data acquisition and real-time basecalling were carried out with MinKNOW software (version 22.05.5). 

#### 2.3.3. Computational Analysis

The raw reads were basecalled using Guppy version 6.1.5 (Oxford Nanopore Technologies, Oxford, UK) in GPU mode using the dna_ r10. 4_ e8.1_ sup. cfg model [21]. The basecalled reads were subsequently filtered to a minimum length of 2 kb and a minimum quality of Q10 using NanoFilt (version 2.8.0) [22]. NanoPlot (version 1.40.0) [22] was used to evaluate the filtered reads. Assembly was performed using Flye (version 2.9-b1778). The quality of the genome assembly was evaluated using QUAST v5.1.0rc1 [23]. Bandage (version 0.8.1) [24] was used to visualize the newly assembled genome of *Fusarium* sp. VM-40 (Figure S1). The draft assembly was subsequently polished in two rounds: first using Racon version 1.4.10 with default settings [25], then Medaka version 0.11.5 with default settings. The completeness of assemblies was evaluated using BUSCO 5.4.3 (ascomycota_odb10 dataset). Genome annotation was carried out using the online platform Genome Sequence Annotation Server (GenSAS, https://www.gensas.org, accessed on 5 Septemper 2022), which provides a pipeline for whole genome structural and functional annotation [26]. The sequencing data and genome assembly for this study have been deposited in the European Nucleotide Archive (ENA) at EMBL-EBI under accession number PRJEB62500.

### 2.4. Comparative Analysis of Fungal Genomes and Phylogenetic Analysis

The whole genome sequence and annotated proteome of 20 other *Fusarium* species, together with *Neonectria ditissima* (to be used as an outgroup), were downloaded from the JGI database and used for phylogenetic and comparative genomics analysis (Table S1).

For the phylogenetic analysis, six barcode sequences were used: the genes coding for the translation elongation factor 1α (*tef1*), RNA polymerase II subunits 1 and 2 (*rpb1* and *rpb2*), and beta-tubulin (*tub2*), as well as the sequence of the internal transcribed spacer (ITS) and the large ribosomal subunit (LSU). These six loci were extracted from each genome, concatenated, and aligned using ClustalW, followed by the generation of a Maximum-Likelihood (ML) tree in IQ-TREE (version 1.6.12) [27] with 1000 bootstrap replicates.

A second phylogenetic tree was built using orthologous proteins. OrthoFinder version 2.5.4 [28] was used to infer phylogeny using predicted protein sequences to determine the phylogenetic relationships. Single-copy orthologous sequences between these species were retrieved, specifying multiple sequence alignment as the method of gene tree inference (-M). The resulting single-copy orthologous sequences were aligned using MAFFT (v7.453) [29] with default parameters. Phylogenetic inferences were conducted using FastTree [30] with local bootstrap values of 1000 replicates. The tree was rooted with *Neonectria ditssima* as an outgroup by the STRIDE algorithm [31].

To better discriminate between the 34 isolates belonging to the *F. tricinctum* species complex, a phylogenetic ML tree was built on the alignment of the *tef1* sequences, which is commonly the first-choice identification marker in *Fusarium* species [32]. Species are listed in Table S2. 

### 2.5. Gene Prediction and Annotation

The tRNA and rRNA were predicted using tRNA scan-SE (version 2.0.11) [33] and barrnap (version 0.9). Gene Ontology (GO) annotation was performed using InterPro (version 66.0) [34]. To predict CAZymes, we used the web-based meta server dbCAN2 [35], which integrates three tools (dbCAN HMM, CAZy, and dbCAN-sub). The three outputs were combined, and CAZymes found by only one tool were removed to improve the CAZyme annotation accuracy. Secondary metabolite biosynthetic clusters were identified using the antiSMASH web server (fungal version 7.0) with the default settings [36].

### 2.6. Extraction of Secondary Metabolites and High-Resolution Liquid Chromatography-Mass Spectrometry (HR-LC-MS) Analysis

Fungal mycelium was transferred to small (⌀ 35 mm × 10 mm) PDA plates supplemented with different concentrations (0, 1, 10, and 100 mM) of the histone deacetylase (HDAC) inhibitor sodium butyrate (SB). The plates were incubated at 25 °C for 14 days alongside empty PDA and PDA-SB plates without the fungus as controls.

For extraction of SMs, the whole agar pads (agar and mycelium) were cut into pieces and transferred to 25 mL glass bottles, then extracted with 4 mL solvent (9:1 ethyl acetate-methanol (*v*/*v*)—0.1% formic acid), spiked with 5 μL caffeic acid standard solution with a concentration of 10 mg·mL^−1^, and sonicated in a sonication bath for one hour. The organic phase was subsequently collected and dried under a gentle stream of N_2_. The dried extracts were resuspended in 500 μL of 1:1 MeOH-MilliQ water (*v*/*v*) and filtered with 0.45 μm PTFE filters.

HR-LC-MS/MS analysis was performed with a Shimadzu Nexera X2 high performance liquid chromatography (HPLC) system with binary LC20ADXR coupled to a Q Exactive Plus hybrid quadrupole-orbitrap mass spectrometer (Thermo Fisher Scientific, Waltham, MA, USA). A Kinetex EVO C18 reversed-phase column was applied for HPLC separations (100 mm × 2.1 mm I.D., 2.6 μm, 100 Å particles, Phenomenex, Torrance, CA, USA), which was maintained at 50 °C. The mobile phase consisted of a gradient of solution A (0.1% formic acid in MilliQ water) and solution B (0.1% formic acid in Acetonitrile). A linear gradient was used: 0–2 min 5% B, 2–17 min linear increase to 50% B, 17–21 min linear increase to 90% B, 21–24 min held at 90% B, 24–24.01 min decrease to 5% B, and 24.01–30 min held at 5% B. The injection volume was 2 µL, and the flow was set to 0.25 mL·min^−1^. MS and MS/MS analyses were performed with electrospray ionization (ESI) in positive mode at a spray voltage of 3.5 kV and sheath and auxiliary gas flow set at 60 and 11, respectively. The ion transfer tube temperature was 300 °C. Spectra were acquired in data-dependent mode with a survey scan at *m*/*z* 100–1500 at a resolution of 70,000, followed by MS/MS fragmentation of the top 5 precursor ions at a resolution of 17,500. A normalized collision energy of 30 was used for fragmentation, and fragmented precursor ions were dynamically excluded for 10 s.

### 2.7. Data Processing and Analysis

The acquired data were further processed by Thermo Scientific FreeStyle software version 1.8. The raw MS/MS data file was converted to mzXML format using the easy convertor provided by the Global Natural Products Social Molecular Networking (GNPS) (https://ccms-ucsd.github.io/GNPSDocumentation/fileconversion/, accessed on 20 March 2023). The data files were subsequently uploaded to GNPS (https://gnps.ucsd.edu/, accessed on 20 March 2023) using WinSCP.

A molecular network was created using the online workflow on the GNPS website [37]. The data were filtered by removing all MS/MS fragment ions within ±17 Da of the precursor *m*/*z*. MS/MS spectra were window filtered by choosing only the top six fragment ions in the ±50 Da window throughout the spectrum. The precursor ion mass tolerance and MS/MS fragment ion tolerance were both set to 0.02 Da. A network was then created where edges were filtered to have a cosine score above 0.7 and more than six matched peaks. Further, edges between two nodes were kept in the network if each of the nodes appeared in the other’s respective top 10 most similar nodes (molecular networking job: https://gnps.ucsd.edu/ProteoSAFe/status.jsp?task=4d9f4cd19b0d4279838c3bea94fa0bff, accessed on 20 March 2023). All mass spectrometry data have been deposited on GNPS under the accession number MassIVE ID: MSV000092159. The molecular network was visualized in Cytoscape version 3.9.1 [38]. Nodes that also existed in the PDA and PDA-SB controls were considered background and thus omitted from the final molecular network. 

The spectra in the network were then searched against the GNPS spectral libraries. Matches were kept with a score above 0.7 and at least six matched peaks. The data was also analyzed by the GNPS molecular library search V2. The precursor ion mass tolerance and fragment ion tolerance were both set to 0.02 Da. The minimal matched peaks were set to six, and the score threshold was 0.7 (library search job: https://gnps.ucsd.edu/ProteoSAFe/status.jsp?task=74d4363bcacf4bcf89db4c4278fd3d73, accessed on 25 March 2023). Several matched annotations in the library search mode were manually added to the molecular network.

## 3. Results and Discussion

### 3.1. Isolate VM-40 from Vinca minor Is a Fusarium

*Vinca minor* is a popular ornamental plant nowadays that was already appreciated by the ancient Romans for its beauty and medicinal properties. It produces a wide array of vinca alkaloids with neuroprotective and antioxidant bioactivities and was used in folk medicine for the treatment of hypertension, as a carminative, emetic, hemostatic, and astringent, and in the treatment of toothache and snakebite [39,40]. In previous studies, three endophytic *Trichoderma* species were isolated from the stems of *V. minor* [41], and ten not further specified species were isolated from various plant tissues [42]. One of these isolates was reported to produce vincamine, the main alkaloid found in *V. minor* leaves [42]. 

In an effort to learn more about the microbes associated with the inner tissues of the *V. minor* plant, we isolated nine endophytic fungi from healthy leaves collected in Groningen, The Netherlands. Based on ITS sequencing, the isolates were identified as *Phialophora* sp., *Pleosporales* sp., *Neocucurbitaria* sp., *Cadophora* sp., *Boeremi* sp., *Lophiostoma* sp., *Alternaria* sp., *Diaporthe* sp., and *Fusarium* sp. The best matches for the *Fusarium* isolate in the NCBI nr/nt database (100% sequence identity) were *F. oxysporum*, *F. tricinctum*, *F. avenaceum*, *F. redolens*, *F. acuminatum*, *F. lateritium*, *F. paeoniae*, *F.* sp., and various uncultured *Fusarium* strains (Dataset S1). This drew our attention since *Fusarium* strains are most known for their plant-pathogenic lifestyle [43], yet the isolate at hand did not cause any visible symptoms of disease. Furthermore, endophytic *Fusarium* species were reported to be a rich source of bioactive compounds, and they have been attracting considerable interest, as recently reviewed by Ahmed et al. [44]. Therefore, we decided to further investigate this fungus in terms of morphology, genomics, and metabolomics.

**Figure 1:**
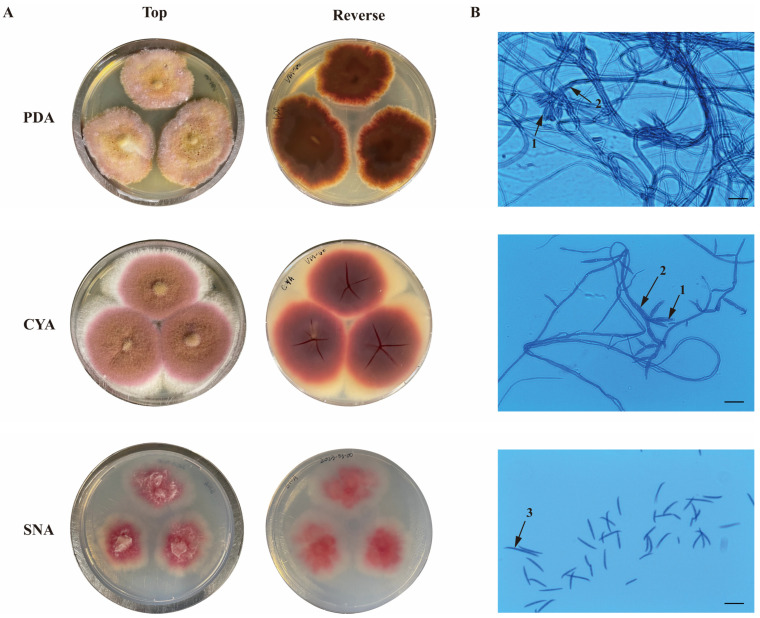
Macroscopic (**A**) and microscopic (**B**) characteristics of *Fusarium* sp. VM-40 on different culture media (PDA, CYA, and SNA), incubated at 25 °C for 7 days. (1) Phialides; (2) Conidiophores; (3) Macroconidia. Scale bars = 25 µm.

The fungal isolate grew on PDA, CYA, and SNA media after 7 days of incubation at 25 °C, spreading with aerial mycelium and smooth, regular margins. On PDA, the colonies attained a diameter of 30–35 mm with a velvety to floccose texture, with a light pink to yellowish color in the front and a dark ruby color in reverse (Figure 1A). Colonies on CYA reached 35–40 mm with dense aerial mycelia and showed a pink coloration with a pale white peripheral border on the obverse side and a yellowish to red color in reverse (Figure 1A). On SNA, *Fusarium* sp. VM-40 formed smaller colonies of 28–32 mm in diameter, with pink coloration in the center and white hyphae at the margin (Figure 1A).

Under the optical microscope, the conidiophores showed branches bearing doliiform phialides. Macroconidia were rare in colonies on PDA and CYA but abundant in colonies on SNA. They were relatively slender, sickle-shaped to almost straight, with 3–5 septae (Figure 1B).

Based on the culture, morphological observation, and ITS regions, the isolated endophytic fungus was preliminary assigned to the genus *Fusarium* and named *Fusarium* sp. VM-40. Since most of its close relatives based on its ITS sequence are uncharacterized fungi, we decided to further investigate its genome.

### 3.2. Genome Sequencing, Assembly, and Genomic Features

With an optimized extraction protocol, we isolated high-quality, high-molecular-weight genomic DNA from the mycelium of *Fusarium* sp. VM-40 (Table S3) and subjected it to long-read sequencing with the Oxford Nanopore Technology. We obtained in total 1,997,205 raw reads (6.1 Gb) with an N50 value of 5.5 kb before filtering and 7.3 kb after filtering with high read quality (Table 1). We assembled the reads into 15 contigs with a total size of 40 Mb and polished the draft assembly by Racon and Medaka. The final assembly revealed a GC content of 47.72% and a BUSCO completeness of 97.4%. Next, we structurally annotated the genome of *Fusarium* sp. VM-40 with GenSAS and predicted 13,546 proteins. For the non-coding RNAs, we predicted 80 rRNAs and 320 tRNAs. Overall, we achieved a highly contiguous assembly (Table S4) with a good degree of completeness. 

### 3.3. Multilocus Phylogeny and Comparative Analysis of the Fusarium sp. VM-40 Genome

**Figure 2:**
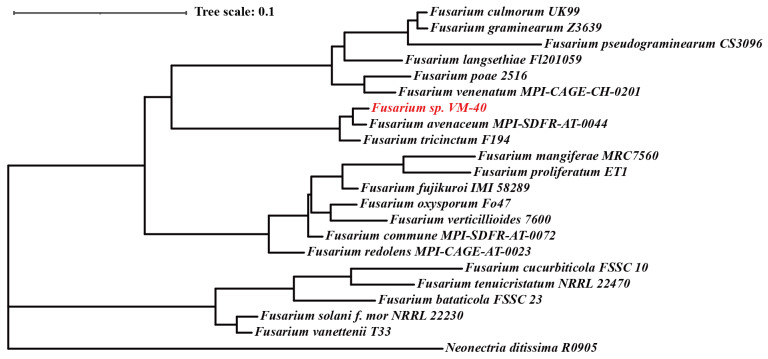
Comparative analysis of the fungal genome and phylogenetic analysis. Maximum-Likelihood phylogenetic tree based on *tef1*, *rpb1*, *rpb2*, *tub2*, ITS, and LSU concatenated nucleotide sequences. *N. ditissima* was used to root the tree. The fungus of interest, *Fusarium* sp. VM-40 is highlighted in red.

In order to continue the taxonomic classification of the fungal isolate, we extracted the sequences of several taxonomic markers from the whole genome assembly and used them for a multilocus phylogenetic analysis. We compared the concatenated sequences of *tef1*, *rpb1*, *rpb2*, *tub2*, ITS, and LSU (~4000 nucleotides in total) of *Fusarium* sp. VM-40 and 20 other *Fusarium* species in the Maximum-Likelihood phylogenetic analysis (including *Neonectria ditssima* as an outgroup) (Figure 2). 

In this analysis, *Fusarium* sp. VM-40 clustered together with *F. avenaceum* and *F. tricinctum*, which both belong to the *F. tricinctum* species complex (FTSC). Due to highly similar barcode sequences across species, taxonomic assignments in a species-rich genus, such as *Fusarium,* can be complicated [45]. To confirm the phylogenetic placement of *Fusarium* sp. VM-40 within the 20 *Fusarium* species, we also performed a whole genome comparison of the 22 species with Orthofinder [28]. Orthofinder identified a total of 316,098 genes in the 22 whole genome sequences and assigned them to 20,601 orthogoups (Dataset S2). From these orthogoups, 6309 orthogoups were shared, 445 were classified as species-specific, and 3613 were single-copy for 22 species. The resulting phylogenetic analysis with 3613 single-copy genes (Figure S2) shows once again that *Fusarium* sp. VM-40 clusters with the FTSC, confirming the previous results.

Finally, we compared the *tef1* sequence of *Fusarium* sp. VM-40 to the sequences of 34 *Fusarium* isolates belonging to the FTSC to further dissect their phylogenetic relationship. We observed that *Fusarium* sp. VM-40 was closely related to *F. paeoniae* and *Fusarium* sp. FTSC 5 (Figure S3). Most species in the FTSC are known plant pathogens. Therefore, we were even more curious to investigate the specific primary and secondary metabolic pathways encoded in the genome of our new *Fusarium* sp. VM-40 in order to find out whether it is likely to be an opportunistic pathogen.

### 3.4. The Genome of Fusarium sp. VM-40 Encodes for Various Enzymes of Biotechnological Interest

**Figure 3:**
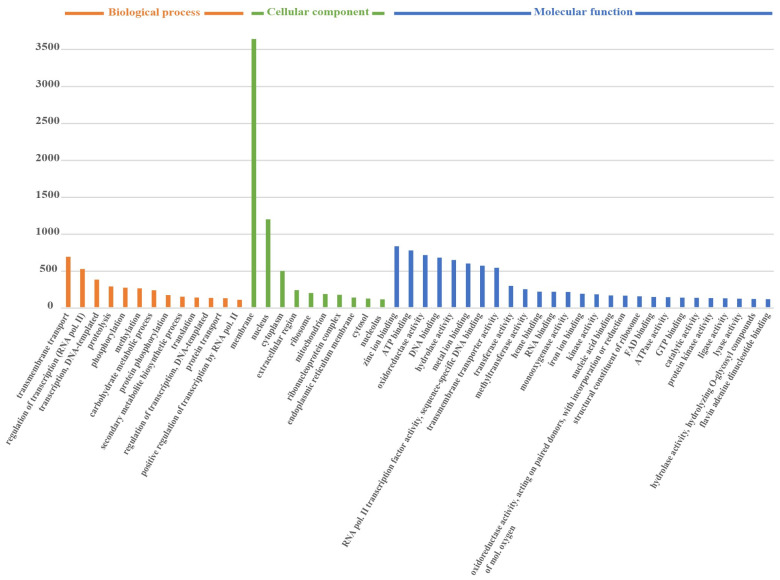
Functional genome annotation of *Fusarium* sp. VM-40 (Top 50 GO terms).

Based on GO annotation, we classified the predicted genes within the *Fusarium* sp. VM-40 genome into functional categories. The top 50 terms were grouped into the three major GO terms as follows: biological processes (18.9%), molecular functions (34.8%), and cellular components (46.3%) (Figure 3).

The gene ontology analysis of the genes that are related to CAZymes includes “carbohydrate metabolic process”, “hydrolase activity”, “hydrolyzing O-glycosyl compounds”, “pectate lyase activity”, and “carbohydrate binding. These enzymes play an important role in carbohydrate degradation, modification, and biosynthesis in fungi and are particularly interesting for industrial applications [46]. In total, dbCAN predicted 691 genes encoding CAZymes in the genome of *Fusarium* sp. VM-40. These could be classified as follows: 313 putative glycoside hydrolases (GHs), 113 putative enzymes with auxiliary activities (AAs), 51 putative carbohydrate esterases (CEs), 114 putative glycosyl transferases (GTs), 29 putative polysaccharide lyases (PLs), and 71 putative enzymes with carbohydrate-binding modules (CBMs). GHs are the predominant type among all the predicted CAZymes of *Fusarium* sp. VM-40. The most abundant (>20 counts) CAZyme types in *Fusarium* sp. VM-40 are GH3 (29), GH43 (27), AA3 (25), GH5 (23), and AA7 (22) (Table S5). In general, when compared with other *Fusarium* species from the CAZy database, this isolate shows a similar abundance of CAZymes [47].

**Figure 4:**
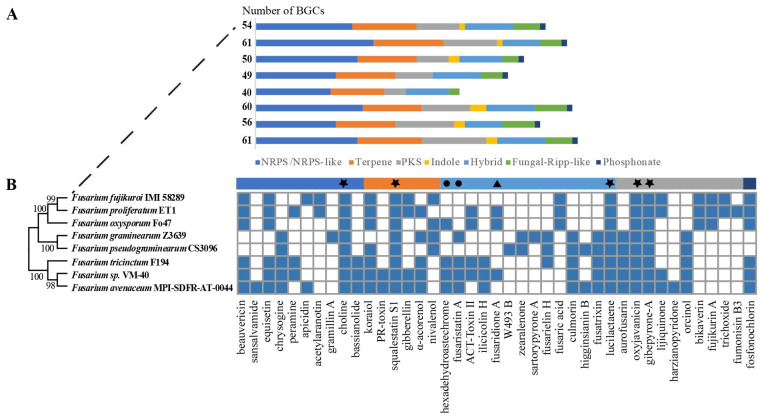
Comparative analysis of predicted BGCs in eight *Fusarium* genomes. (**A**) Number of BGCs in each genome by BGC type; (**B**) Phylogenetic tree of the analyzed species constructed based on the *tef1* gene next to the presence (blue) and absence (white) of 30 known BGCs in the eight *Fusarium* genomes. BGCs discussed in the text are highlighted with symbols. Star: BGCs predicted to give rise to same SMs across all genomes; Circle: BGCs with potential biocontrol function; Triangle: BGC only present in *Fusarium* sp. VM-40 but not in other FTSC strains.

Although not essential for life, secondary metabolism is an important biological process, e.g., for niche adaptation, inter- and intra-species communication, and competition. Therefore, we queried the genomes of *Fusarium* sp. VM-40 and seven *Fusarium* species from Table S1 for biosynthetic gene clusters (BGCs) using the online-based tool fungiSMASH [36]. *Fusarium* sp. VM-40 possesses 56 BGCs for secondary metabolite biosynthesis, classified as follows based on the class of core biosynthetic enzymes: 12 polyketide synthases (PKSs), 15 NRPSs (nonribosomal peptide synthetases), 6 NRPS-PKS hybrids, 1 indole, 1 NRPS-indole hybrid, 11 terpene synthases (TSs), 1 NRPS-TS, 6 fungal-ribosomally synthesized and post-translationally modified peptides (RiPPs), and one phosphonate cluster (Figure 4A, Table S6). Among them, 25 BGCs showed similarities with known BGCs in the MiBIG database [48] and are predicted to give rise to various types of compounds (Figure S4). 

Across all *Fusarium* species analyzed, we predicted between 40 and 61 BGCs, averaging 47 clusters per genome. In total, 41 clusters showed similarities with known BGCs in the MiBIG database. Among the predicted clusters, NRPS clusters are the most abundant type of BGC, followed by terpene, PKS, and hybrid clusters, in particular NRPS-T1PKS hybrids. We constructed a presence/absence matrix of these known BGCs to visualize the biosynthetic diversity among the eight *Fusarium* genomes (Figure 4B). The comparison revealed considerable differences with only five BGCs, namely the ones predicted to give rise to choline, squalestatin S1, lucilactaene, oxyjavanicin, and gibepyrone A, conserved across all genomes. *Fusarium* sp. VM-40 shares 19 out of the 30 known BGCs with *F. avenaceum and F. tricinctum*. In addition, *Fusarium* sp. VM-40 shares a hexadehydroastechrome (HAS) BGC with these two species and *F. oxysporum* Fo47. *F. oxysporum* Fo47 is a putative biocontrol strain, and the HAS cluster has been implicated in this biocontrol function since it is absent in pathogenic strains of the same species, such as *F. oxysporum f.*sp. *lycopersici*, which is pathogenic to tomatoes [49]. In *Fusarium* sp. VM-40, the core NRPS gene of the HAS BGC shows 73% sequence identity with that in *A. fumigatus* [50], yet it remains unclear what the role of the HAS cluster is in these FTSC isolates. 

Another BGC shared among *Fusarium* sp. VM-40, *F. avenaceum*, and *F. tricinctum* is also present in the *F. graminearum* Z3639 genome. It is predicted to give rise to fusaristatin A, the in vivo function of which is unknown. Interestingly, however, previous research has suggested that the fusaristatin A BGC is only present in a subset of Western Australian *F. pseudograminearum* isolates [51], and its absence appears to be associated with increased crown rot aggressiveness of *F. pseudograminearum* on wheat [52]. This implies that fusaristatin A also has a biocontrol function.

The BGC predicted to produce fusaridione A is only present in *Fusarium* sp. VM-40 within FTSC. The core gene of this BGC shows 58% sequence identity with that in *Fusarium heterosporum,* in which fusaridione A was first isolated [53]. The biosynthesis and biological functions of fusaridione A are thus far unknown, which is likely due to the fact that this compound is highly unstable.

Taken together, the number of predicted BGCs in *Fusarium* sp. VM-40 indicates that this species has a broad potential for SM biosynthesis and is worth further analysis.

### 3.5. Fusarium sp. VM-40 Produces a Wide Range of Secondary Metabolites

**Figure 5:**
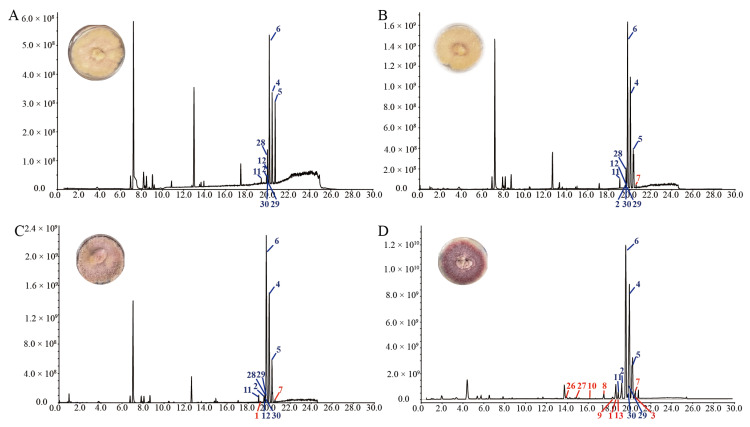
TICs chromatograms in positive ion mode of EtOAc extracts of *Fusarium* sp. VM-40 grown in the absence and presence of different concentrations of SB. (**A**) *Fusarium* sp. VM-40 control group; (**B**) *Fusarium* sp. VM-40 grown on medium with 1 mM SB; (**C**) *Fusarium* sp. VM-40 grown on medium with 10 mM SB; (**D**) *Fusarium* sp. VM-40 grown on medium with 100 mM SB at 25 °C for 14 days. Tentatively assigned peaks are labeled in red (only detected upon SB treatment) and blue (detected in all treatment groups).

A preliminary analysis of the crude organic extracts from cultures of *Fusarium* sp. VM-40 showed poor production of SMs (Figure 5A), despite the relatively high abundance of BGCs. This suggests that many of these clusters are expressed at low levels or are completely silent under standard culture conditions. To overcome this, we set up epigenetic manipulation experiments. We grew *Fusarium* sp. VM-40 on media containing 1, 10, and 100 mM sodium butyrate, a commonly used epigenetic modulator, monitored the phenotype of the cultures, and analyzed the TIC chromatograms of crude extracts after 14 days of cultivation (Figure 5).

With increasing concentrations of SB, the fungal colony gradually turns from yellow to red in morphology, indicating that new metabolites are produced, possibly due to one or more BGCs being upregulated by the effect of SB in a dose-dependent fashion. As expected, we also observed several changes in the TIC: the abundance of several peaks with retention times (rt) around 20 min, labeled **2**, **4, 5**, **6**, and **11** in Figure 5A, gradually increases upon treatment with increasing concentrations of SB. Peak **28**, however, and others remain unchanged in the treatment groups. There are also several new peaks that appear in the extracts of fungus grown in the presence of 1 and 10 mM SB (Figure 5B,C), e.g., the small peaks **1** and **7** (rt around 20 min), and the peaks *m*/*z* 607.3800 (rt 14.08 min), and *m*/*z* 639.4057 (rt 15.83 min). Interestingly, these latter, unidentified peaks are again absent in the extracts of the fungus grown in the presence of 100 mM SB (Figure 5D). In addition to the thus far discussed changes, treatment with 100 mM SB also elicits the production of more new compounds, labeled as **3**, **8**, **9**, **10**, **13**, **26**, **27** in Figure 5D, as well as the unidentified peaks *m*/*z* 625.3956 (rt 11.65 min), *m*/*z* 483.3202 (rt 13.01 min), *m*/*z* 414.1929 (rt 13.43 and 13.89 min), *m*/*z* 432.2036 (rt 13.95 min), *m*/*z* 416.2086 (rt around 15 min), and *m*/*z* 567.3784 (rt 16.73 min).

**Figure 6:**
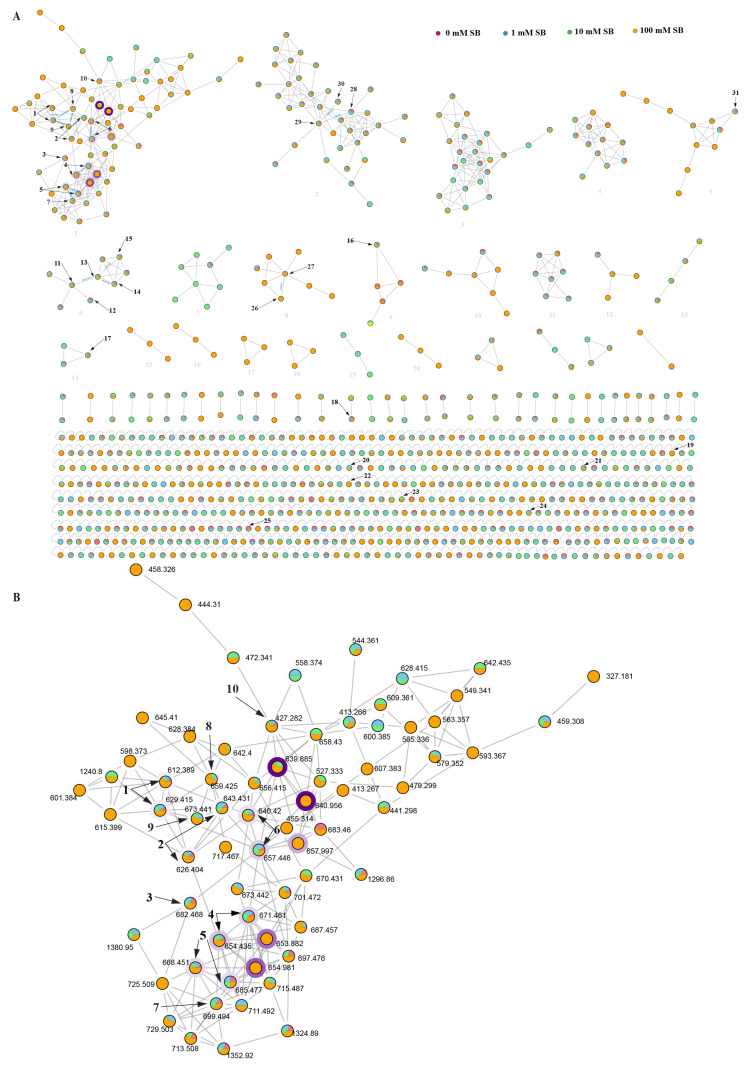
Molecular network of *Fusarium* sp. VM-40 secondary metabolites in the presence and absence of different concentrations of SB. (**A**) Overview of the molecular network, with the clusters numbered in blue and the identified compounds in black; (**B**) Molecular network of the enniatins (cluster 1). Node colors indicate the presence of the respective compound in the 0 mM SB (red), 1 mM SB (blue), 10 mM SB (green), and 100 mM SB (orange) samples. Darker shades of node borders indicate higher metabolite abundance in samples based on peak area integration of the base peak.

To gain further information on the chemical diversity of the *Fusarium* sp. VM-40 metabolome, especially the differentially produced SMs, we performed a molecular networking analysis using the Global Natural Products Social Molecular Networking (GNPS) platform. The generated molecular network was manually curated by deleting nodes present in the PDA and PDA-SB control groups, ultimately leading to a molecular network consisting of 895 nodes (Figure 6, Dataset S3). Each node is represented as a pie chart, where different colors correspond to secondary metabolites that exist in groups with different concentrations of SB in the medium. The border width of the node indicates the relative abundance of the compounds in the extract. 

**Figure 7:**
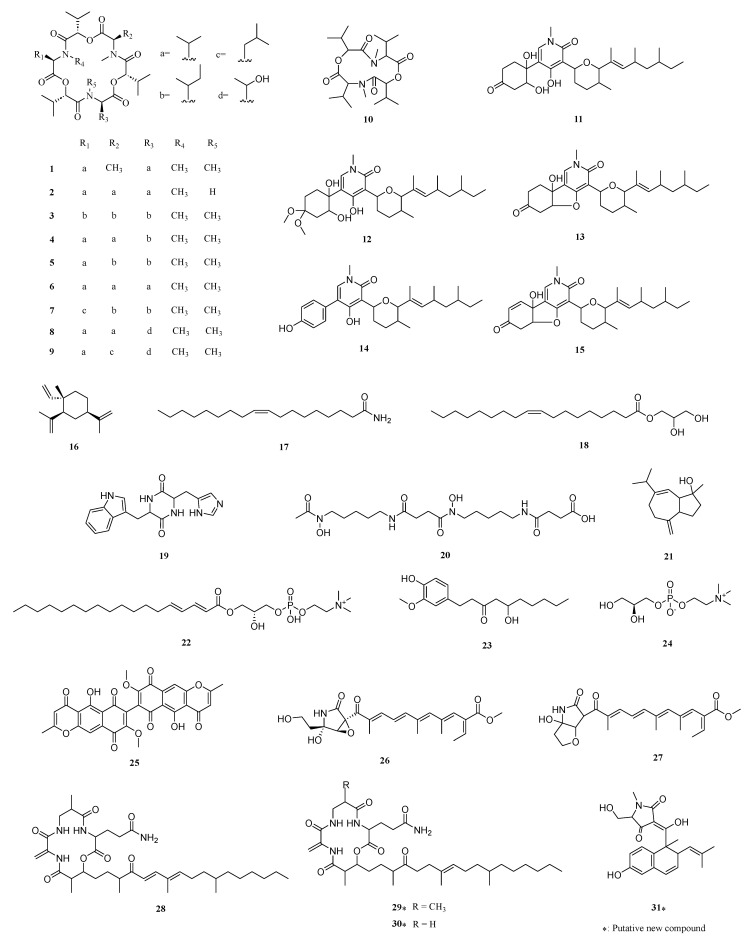
Chemical structures of the secondary metabolites identified in the crude extracts of *Fusarium* sp. VM-40.

Within this network, we identified 31 compounds either via a direct match with the GNPS MS/MS spectral library or via inference from matched adjacent nodes (Table 2, Figure 6 and Figure 7). For instance, we identified the major constituents of the extracts with rt around 20 min to be enniatins (ENNs). ENNs are cyclic hexadepsipeptides consisting of alternating N-methyl amino acids and hydroxy acid residues [54]. The most abundant metabolites in the *Fusarium* sp. VM-40 extract are enniatin B (**6**) and enniatin B1 (**4**), with ions [M + H]^+^ and [M + NH_4_]^+^ (Figure 5A and Figure 6B). Upon treatment with SB, they become even more abundant, and highly similar compounds occur with near identical rt and product ions. Analogously, *m*/*z* 612.389 and *m*/*z* 629.415 are predicted to be the [M + H]^+^ and [M + NH_4_]^+^ adducts of enniatin J1 (**1**). Enniatin J1 (**1**) itself is found in the 0 mM, 1 mM, and 10 mM SB groups in trace amounts, whereas a distinct peak is observed in the 100 mM SB group. According to the molecular network, compound **8** (*m*/*z* 659.425) shows a -CH_2_O difference with **1**. According to the fragmentation rules of ENNs reported in the literature [54], we speculate that **8** is enniatin P1. Interestingly, the amount of **8** (rt 17.54 min) significantly increased with the treatment of 100 mM SB, indicating that SB might influence the expression of the enniatin BGC in region 9.1 in the *Fusarium* sp. VM-40 genome. Similarly, compound **9** (*m*/*z* 673.441) showed -C_2_H_4_O and -CH_2_ differences with **1** and **8**, respectively. It is formed by 2-hydroxy isovaleric acid (Hiv), aliphatic N-methyl-valine (NMeVal), N-methyl-threonine (NMeThr), and N-methyl-leucine (NMeLeu), which is proposed to be enniatin P2. Compound **9** is only present in trace amounts in the 1 mM and 10 mM SB groups, while in the 100 mM SB group, a distinct small peak of **9** (rt 18.30 min) is detected. The *m*/*z* 626.404 and *m*/*z* 659.425 are predicted as enniatin B2 (**2**).

Which consists of Hiv and NMeVal. Similarly, trace amounts of compound **2** are detected in 0 mM, 1 mM, and 10 mM SB groups, while after 100 mM SB treatment, a higher peak at a retention time of 19.30 min appears. Compound **3** (*m*/*z* 682.468) is predicted as enniatin A, which is composed of Hiv and N-methyl-isoleucine (NMeIle). In the MS/MS spectrum, compound **3** possesses a fragment ion at *m*/*z* 100.1125 as a result of y_NMeLeu/Ile_, and the lack of fragment ion y_NMeVal_ at *m*/*z* 86.0967 also demonstrates the absence of the isopropyl group. Compound **5** (*m*/*z* 668.451 and *m*/*z* 685.477) is predicted as enniatin A1. Compared with compound **3**, compound **5** has a NMeVal group instead of NMeIle, which contributes to the discovery of the fragment ion y_NMeVal_ (*m*/*z* 86.0967). In addition, according to the molecular network, the node at *m*/*z* 699.494 (**7**) is related to **5**, with a -CH_5_N group difference. In the MS/MS spectrum of compound **7**, only one fragment ion at *m*/*z* 100.1125 is found, indicating that compound **7** possesses NMeLeu or NMeIle but not NMeVal. It is worth mentioning that **3** and **7** do not display a direct association with each other in the molecular network. Combined with the reference [13], compound **7** is predicted to be enniatin F, formed by three Hiv groups, two NMeIle groups, and one NMeLeu group. Compound **7** also exists in all SB treatment groups, becoming more abundant with increasing SB concentrations.

In addition to the cyclic hexadepsipeptides mentioned above, we also find a cyclic tetradepsipeptide **10** (*m*/*z* 427.282) (Figure 7 and Figure S6). The fragment ion *m*/*z* 86.0967 indicates the existence of y_NMeVal_, and we speculate that the ions of *m*/*z* 314.1976 and *m*/*z* 214.1449 are [M + H-NMeVal]^+^ and [M + H-NMeVal-Hiv]^+^. 

We further identified a cluster of pyridine-type amides in the molecular network (cluster 6). Oxysporidinone (**11**) and its dimethyl-ketal derivative (**12**) show high peaks in the extracts of fungi grown in the presence of 0 mM, 1 mM, and 10 mM SB. However, upon addition of 100 mM SB, **12** is not detected, and only **11** is present in the extract. Compound **13** shows a -H_2_O group difference compared with **11**, and could therefore be 4, 6′-anhydrooxysporidinone. Analogously, **14** and **15** are predicted to be sambutoxin, and (E)-4-(6-(4,6-dimethyloct-2-en-2-yl)-5-methyltetrahydro-2H-pyran-2-yl)-9a-hydroxy-2-methyl-2,5a,6,9a-tetrahydrobenzofuro [3,2-c]pyridine-3,7-dione.

Compound **26** from cluster 8 is annotated by GNPS as fusarin C. Based on the mass difference, we speculate that compound **27** is lucilactaene. Moreover, most of the 2-pyrrolidone derivates in cluster 8 could only be found in the 100 mM SB group, indicating that 100 mM SB might trigger expression of the BGC in region 10.4, which may be responsible for forming lucilactaene analogs. It was reported that lucilactaene and its derivatives could be promising lead compounds for antimalarial drug development because of their their unique structure [55].

Our in silico analysis of the *Fusarium* sp. VM-40 genome (Figure 4) indicates that this fungus has a BGC to produce compounds related to fusaristatin A (region 14.3). Although no such compounds were initially annotated by GNPS, we identified compound **28** in cluster 2 as fusaristatin A according to its MS2 fragmentation pattern. Compound **29** in the same cluster shows a 2H difference with **28,** and we predict it to be (E)-3-(6,13-dimethyl-10-methylene-2,5,9,12-tetraoxo-14-(3,7,11-trimethyl-4-oxoheptadec-7-en-1-yl)-1-oxa-4,8,11-triazacyclotetradecan-3-yl)propenamide. The *m*/*z* of compound **30** suggests an elimination of a -CH_2_ group compared with compound **29**, and it is predicted to be (E)-3-(13-methyl-10-methylene-2,5,9,12-tetraoxo-14-(3,7,11-trimethyl-4-oxoheptadec-7-en-1-yl)-1-oxa-4,8,11-triazacyclotetradecan-3-yl)propenamide. Therefore, we conclude that cluster 2 represents a group of cyclic lipopeptides.

In addition to the identified secondary metabolites, there are several other visible peaks at *m*/*z* 314.3400, *m*/*z* 607.3800, *m*/*z* 639.4057, *m*/*z* 625.3956, *m*/*z* 414.1929, and *m*/*z* 432.2036, that have no matched annotations and are still unknown. These compounds need to be further explored through chemical isolation and structural characterization.

Overall, the addition of 100 mM sodium butyrate to the cultivation medium significantly altered the metabolic profile of *Fusarium* sp. VM-40, resulting in increased production of known compounds and several putative new compounds. Several other nodes in the molecular network could not be assigned to any known compound, suggesting that these might be novel metabolites that need further investigation.

### 3.6. Metabologenomic Analysis—Linking Secondary Metabolites to BGCs of Fusarium sp. VM-40

**Figure 8:**
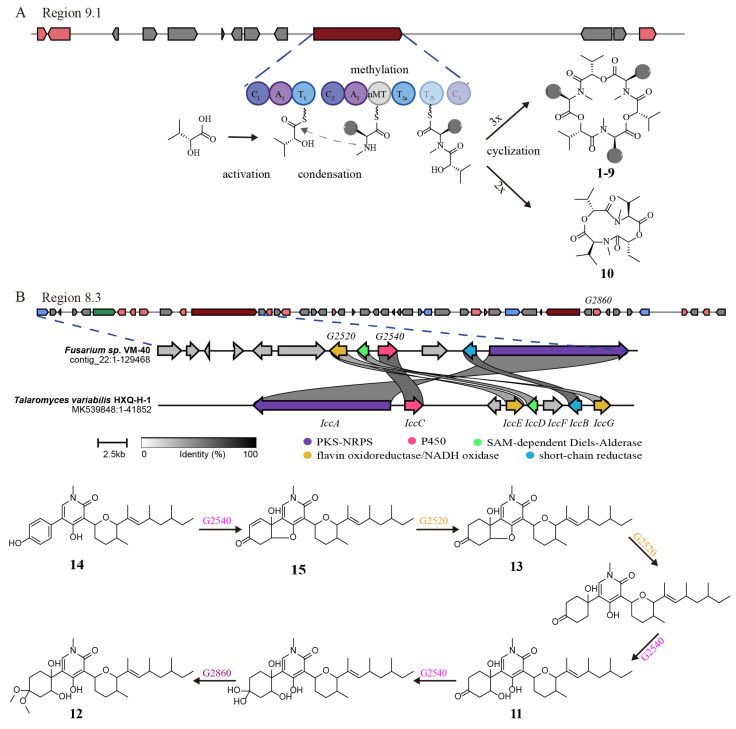
(**A**) Proposed biosynthetic pathway of enniatins and compound **10**. (**B**) Proposed biosynthetic pathway for oxysporidinone analogs of **11**–**15**. Comparison of the biosynthetic gene cluster *icc* in *Talaromyces variabilis* HXQ-H-1 (MK539848) and region 8.3 in *Fusarium* sp. VM-40 by using clinker [68].

Based on the genomic and metabolomic analyses above, it shows that *Fusarium* sp. VM-40 has the potential to produce a diverse set of SMs. Among them, the most striking group were the enniatins. ENNs are cyclic hexadepsipeptides formed by the condensation of three D-α-hydroxy acids and three N-methyl-L-amino acids. Many of the biological activities of the enniatins are of pharmaceutical interest, such as antimicrobial activities [56], inhibitors of major drug efflux pumps [57], and acyl-CoA cholesterol acyltransferase inhibition [58]. The structural differences related to the N-methyl-amino acids were previously linked to the different bioactivities of the ENNs [59]. In this study, we identified nine enniatin analogs (compounds **1**–**9**) with the highest amount of enniatin B (**6**), followed by enniatin B1 (**4**) and enniatin A1 (**5**) [60]. The main amino acid constituents in these compounds are N-methyl-valine, N-methyl-isoleucine, and N-methyl-leucine. However, we also identified two N-methyl-threonine-containing compounds, enniatin P1 (**8**) and enniatin P2 (**9**). These compounds were only produced upon stimulation with 100 mM SB, and to our knowledge, there are no prior studies on their bioactivities. Therefore, they could be interesting candidates for future investigations. The BGC in region 9.1 (NRPS) of the *Fusarium* sp. VM-40 genome is predicted to be responsible for the production of ENNs. As shown in Figure 8A, the A1 domain of the first module activates the D-2-hydroxycarboxylic acid substrate and loads it onto the T1 domain in the same module. The A2 domain of the second module activates and loads an L-amino acid substrate molecule onto each of the adjacent twin T2 domains. Amide bond formation between the D-2-hydroxycarboxylic acid and N-methyl-L-amino acid thioesters is carried out by the C2 domain. This generates the dipeptidol monomer, three or four copies of which would then be ligated and finally cyclized in a programmed cyclooligomerization process to generate the cyclohexadepsipeptide or cyclooctadepsipeptide products, respectively [60]. However, the cyclization of two dipeptidol monomers into a cyclic tetradepsipeptide has not been reported yet.

Interestingly, we identified one such cyclic tetradepsipeptide, compound **10**, which was only detected in extracts of fungi challenged with 100 mM SB. A similar compound [-(aoxyisohexanoyl-N-methyl-Leu)2-] was first isolated from the endophytic fungus *F*. *tricinctum* SYPF 7082 of *Panax notoginseng* [61]. The novel structure of **10** makes it an interesting candidate for investigating its bioactivities and biosynthesis, which may rely on the same NRPS as the cyclohexadepsipeptides (Figure 8A). 

Compounds **11**–**13** were previously isolated from *F*. *oxysporum*, and compound **11** shows antifungal effects [62,63]. Moreover, the structures of these compounds are similar to ilicicolin H, and region 8.3 in the BGC of this fungus might be responsible for their biosynthesis. This gene cluster mainly contains genes encoding a central PKS-NRPS hybrid, a PKS, one sugar transport protein, a serine/threonine protein kinase, an NADH: flavin oxidoreductase/NADH oxidase, two methyltransferases, two cytochromes P450, a crotonyl-CoA reductase/alcohol dehydrogenase, a nitrilase/cyanide hydratase, and apolipoprotein.

A recent study identified two enzymes (OsdM and OsdN) involved in the phenol dearomatization process in the formation of oxysporidinone [64]. Based on the similarity of the PKS-NRPS and the P450s to those in the reported oxysporidinone biosynthesis gene cluster, we propose that compounds **11**–**15** follow a similar biosynthetic route (Figure 8B). At the same time, region 8.3 bears a significant number of unknown genes, and thereby potentially interesting bioactive compounds need to be further explored.

The highly interesting compounds **26**–**27** and **28**–**30** are biosynthesized by enzymes encoded in T1PKS-NRPS hybrid BGCs (regions 10.4 and 14.3) and have attracted the interest of researchers due to their unique structures. The biosynthetic gene cluster of lucilactaene (**27**) was identified in *Fusarium* sp. RK 97–94, and a putative biosynthetic pathway was proposed [65,66]. The biosynthetic gene cluster for fusaristatin A (**28**) was identified in *F. graminearum* and partially characterized [67]. Future studies could further elaborate on the biosynthesis pathway of these interesting compounds.

## 4. Conclusions

*Fusarium* is a treasure trove of SMs with diverse chemical structures and biological properties. In addition to phylogenetic analysis based on the multi-locus and whole-genome sequence, we obtained a high-quality whole-genome sequence of the endophytic strain *Fusarium* sp. VM-40 from *Vinca minor* and extensively analyzed it by gene prediction and annotation in this work. Our initial morphological characterization and ITS-based identification were sufficient to categorize *Fusarium* sp. VM-40 as a *Fusarium* species. A six-locus gene tree (*tef1*, *rpb1*, *rpb2*, *tub2*, ITS, and LSU) and a phylogenetic analysis based on single-copy orthologs with 21 *Fusarium* species showed that *Fusarium* sp. VM-40 is clustered together with *Fusarium avenaceum* and *Fusarium tricinctum*, which are both from the FTSC. Further, a phylogenetic analysis based on *tef1* with 34 FTSC isolates revealed that *Fusarium* sp. VM-40 is closely related to *Fusarium paeoniae.* Within the *Fusarium* sp. VM-40 genome, we predicted various BGCs, two of which were previously implicated in the biocontrol properties of *Fusarium* species. For one of these, the fusaristatin A BGC, we even identified several potential pathway products in the extracts of *Fusarium* sp. VM-40. This observation may open the door for further investigation of this fungal isolate to elucidate its biological function within the endophytic microbiome.

Our chemical investigation of the fungal extract further indicated that the most abundant SMs produced by *Fusarium* sp. VM-40 under standard culture conditions are cyclic depsipeptides. Therefore, a great number of other types of BGCs in this strain are silent and/or expressed at a low level. In the current study, to explore its potential for producing metabolites, an epigenetic manipulation strategy using an HDAC inhibitor was employed to activate cryptic BGCs. Remarkably, our metabolomic analysis reveals a large diversity of metabolic changes and allows the identification of some potentially new compounds upon treatment with 100 mM sodium butyrate. Therefore, these findings open possibilities for targeted genome mining, such as gene knockout, introduction or heterologous expression of microbial genes, regulation of promoters, and induction of mutations to biosynthesize newer bioactive SMs for new drug research and development.

## Figures and Tables

**Table 1:** Read and assembly statistics of *Fusarium* sp. VM-40.

	Unfiltered	Filtered (>2 kb)
No. of bases (Gb)	6.1	5.0
No. of reads	1,997,205	866,843
N50 (kb)	5.5	7.3
Mean read length (bp)	3060.1	5784.4
Mean read quality	15.9	17.4
Coverage	100×	
Polishing steps	Racon + Medaka	
No. of contigs (≥50,000 bp)	15	
Genome size (Mb)	40	
GC (%)	47.72	
BUSCO (Ascomycota_odb10) (%)	97.4	
Number of the protein-coding genes	13,546	
tRNA genes	320	
rRNA genes	80	
Proteins with a predicted Pfam domain	11,402	
Proteins with CAZymes proteins	691	

**Table 2:** Identification of metabolites in *Fusarium* sp. VM-40 before and after sodium butyrate treatment by the molecular network.

Number	Metabolite Name	rt (min)	Experimental Mass (*m*/*z*)	Adduct	Molecular Formula	Molecular Weight	Theoretical Mass (*m*/*z*)	Mass Error (ppm)	MS2 Fragment Ions (*m*/*z*)	Samples			
										0 mM ^a^	1 mM ^b^	10 mM ^c^	100 mM ^d^
1	Enniatin J1	20.23/20.18/20.16/18.67	612.3829/612.3835/612.3834/612.3887	[M + H]^+^	C_31_H_53_N_3_O_9_	611.78	612.3866	−5.97/−4.99/−5.15/3.50	499.3041, 399.2508, 286.1663, 214.1449, 196.1343, 186.1135, 168.1028, 86.0967	t	t	+	+
		20.22/20.17/20.24/18.67	629.4094/629.4097/629.4100/629.4146	[M + NH_4_]^+^			629.4131	−5.88/−5.41/−4.93/2.38					
2	Enniatin B2	20.56/20.60/20.48/19.21	626.3988/626.3989/626.3996/626.4046	[M + H]^+^	C_32_H_55_N_3_O_9_	625.80	626.4022	−5.43/−5.27/−4.16/3.83	513.3196, 413.2675, 314.1979, 214.1449, 196.1344, 186.1500, 86.0968	+	+	+	+
		20.54/20.50/20.47/19.30	643.4251/643.4254/643.4252/643.4313	[M + NH_4_]^+^			643.4288	−5.68/−5.21/−5.52/3.96					
3	Enniatin A	21.66/21.60/21.57/20.51	682.4611/682.4611/682.4614/682.4680	[M + H]^+^	C_36_H_63_N_3_O_9_	681.91	682.4648	−5.43/−5.43/−4.99/4.68	555.3663, 455.3137, 328.2134, 228.1606, 210.1499, 200.1656, 100.1125	t	t	t	+
4	Enniatin B1	21.24/21.01/21.07/19.96	671.4566/671.4568/671.4557/671.4609	[M + NH_4_]^+^	C_34_H_59_N_3_O_9_	653.86	671.4601	−5.14/−4.84/−6.48/1.26	541.3507, 441.2983, 328.2135, 314.1975, 228.1606, 214.1448, 196.1343, 186.1498, 100.1125, 86.0967	+	+	+	+
		21.06/21.02/21.18/19.91	654.4300/654.4301/654.4302/654.4352	[M + H]^+^			654.4335	−5.35/−5.20/−5.09/2.59					
5	Enniatin A1	21.34/21.29/21.45/20.25	668.4450/668.4463/668.4456/668.4514	[M + H]^+^	C_35_H_61_N_3_O_9_	667.89	668.4491	−6.21/−4.27/−5.32/3.36	541.3527, 441.2988, 328.2134, 314.1977, 228.1606, 210.1500, 196.1343, 100.1125, 86.0967	+	+	+	+
		21.51/21.46/21.35/20.22	685.4721/685.4723/685.4711/685.4771	[M + NH_4_]^+^			685.4757	−5.26/−4.96/−6.72/2.04					
6	Enniatin B	20.79/20.76/20.73/19.58	657.4408/657.4412/657.4412/657.4459	[M + NH_4_]^+^	C_33_H_57_N_3_O_9_	639.83	657.4444	−5.48/−4.87/−4.87/2.27	587.0684, 527.3361, 314.1976, 214.1449, 196.1343, 186.1499, 86.0967	+	+	+	+
		20.79/20.76/20.73/19.56	640.4142/640.4145/640.4146/640.4203	[M + H]^+^			640.4179	−5.71/−5.24/−5.08/3.82					
7	Enniatin F	21.60/21.62/21.62/20.45	699.4878/699.4882/699.4880/699.4939	[M + NH_4_]^+^	C_36_H_63_N_3_O_9_	681.91	699.4914	−5.08/−4.51/−4.79/3.64	555.3675, 455.3140, 328.2133, 228.1606, 210.1500, 200.1655, 182.1548, 100.1125	t	+	+	+
8	Enniatin P1	19.55/17.54	659.4203/659.4255	[M + NH_4_]^+^	C_32_H_55_N_3_O_10_	641.80	659.4237	−5.11/2.78	642.3979, 624.3943, 529.3160, 511.3055, 429.2618, 411.2516, 314.1987, 298.1658, 214.1449, 196.1343, 180.1029, 154.0871, 86.0968	-	-	t	+
9	Enniatin P2	19.98/20.06/18.30	673.4358/673.4359/673.4421	[M + NH_4_]^+^	C_33_H_57_N_3_O_10_	655.83	673.4393	−5.22/−5.08/4.13	656.4175, 610.4143, 556.3628, 511.3056, 425.2678, 298.1663, 210.1500, 196.1344, 100.1125, 86.0968	-	t	t	+
10	3,6,9,12-tetraisopropyl-4,10-dimethyl-1,7-dioxa-4,10-diazacyclododecane-2,5,8,11-tetraone	18.18/18.12/18.09/16.15	427.2787/427.2791/427.2788/427.2823	[M + H]^+^	C_22_H_38_N_2_O_6_	426.55	427.2814	−6.23/−5.29/−5.99/2.205	314.1976, 214.1449, 186.1498, 86.0967	t	t	t	+
11	Oxysporidinone	20.07/20.05/20.05/18.97	490.3148/490.3143/490.3145/490.3190	[M + H]^+^	C_28_H_43_NO_6_	489.65	490.3174	−5.33/−6.35/−5.94/3.24	472.3093, 454.2981, 436.2867, 274.1088, 256.0981, 230.0824, 123.1174	+	+	+	+
12	Dimethyl ketal of oxysporidinone	20.70/20.66/20.66	536.3564/536.3562/536.3561	[M + H]^+^	C_30_H_49_NO_7_	535.72	536.3593	−5.36/−5.74/−5.92	468.3091, 450.2992, 338.1736, 288.1230, 312.1579, 270.1112, 244.0954, 232.0956	t	t	t	-
13	4,6′-Anhydrooxysporidinone	19.95/19.91/20.08/18.84	472.3043/472.3041/472.3043/472.3081	[M + H]^+^	C_28_H_41_NO_5_	471.64	472.3068	−5.39/−5.82/−5.39/2.65	472.3089, 454.2981, 436.2873, 342.1718, 248.0931, 230.0825	t	t	t	t
14	Sambutoxin	20.99/20.98/20.96/20.13	454.2935/454.2934/454.2936/454.2971	[M + H]^+^	C_28_H_39_NO_4_	453.62	454.2963	−6.12/−6.34/−5.90/1.80	436.2828, 324.1573, 298.1418, 256.0955, 230.0800, 218.0806, 175.1473, 137.1315, 123.1161, 109.1006, 95.0850,	t	t	t	t
15	(E)-4-(6-(4,6-dimethyloct-2-en-2-yl)-5-methyltetrahydro-2H-pyran-2-yl)-9a-hydroxy-2-methyl-2,5a,6,9a-tetrahydrobenzofuro[3,2-c]pyridine-3,7-dione	20.18/20.16/20.16/19.24	470.2885/470.2882/470.2885/470.2921	[M + H]^+^	C_28_H_39_NO_5_	469.62	470.2912	−5.73/−6.37/−5.73/1.92	452.2769, 340.1533, 314.1364, 312.1214, 272.090, 246.0748, 228.0638, 137.1320, 109.1107, 95.0850, 69.0696	t	t	t	t
16	Beta-elemene	15.09/15.05/15.02/13.09	205.1943/205.1944/205.1944/205.1960	[M + H]^+^	C_15_H_24_	204.36	205.1962	−9.13/−8.65/−8.65/−0.85	149.1317, 135.1160, 121.1004, 109.1004, 95.0849	t	t	t	t
17	9-(Z)-octadecenamide	21.40/21.40/21.39/20.79	563.5490/563.5490/563.5490/563.5538	[2M + H]^+^	C_18_H_35_NO	281.48	563.5521	−5.51/−5.51/−5.51/3.01	282.2775, 265.2510, 247,2409, 135.1160, 97.1006, 83.0850, 69.0696	t	t	t	t
18	Monoolein	21.39/21.36/20.65	357.2981/357.2981//357.3013	[M + H]^+^	C_21_H_40_O_4_	356.55	357.3010	−8.21/−8.21/0.75	339.2882, 265.2509, 247.2408, 177.1627, 149.1317, 135.1161, 121.1006, 95.0850, 83.0851, 69.0696, 57.0699	t	t	-	t
19	3-(1H-imidazol-4-ylmethyl)-6-(1H-indol-3-ylmethyl)-2,5-piperazinedione	1.74	324.1471	[M + H]^+^	C_17_H_17_N_5_O_2_	323.36	324.1466	1.55	195.0888, 159.0928, 130.0661, 110.0717, 71.4404	-	-	-	t
20	3,14-dihydroxy-2,10,13,21-tetraoxo-3,9,14,20-tetraazatetracosan-24-oic acid	19.37/19.34	478.2910/478.2902	[M + NH_4_]^+^	C_20_H_36_N_4_O_8_	460.53	478.2882	5.78/4.10	337.2721, 175.1486, 95.0851, 69.0697	-	t	t	-
21	1-methyl-4-methylidene-7-(propan-2-yl)-1,2,3,3a,4,5,6,8a-octahydroazulen-1-ol	13.57/13.52/13.48/13.11.36	203.1787/203.1787/203.1786/203.1803	[M-H_2_O + H]^+^	C_15_H_24_O	220.36	203.1805	−8.98/−8.98/−9.47/−1.10	161.1314, 147.1161, 133.1006, 117.0692, 109.1003, 95.0850, 83.0852, 69.6548	t	t	t	t
22	PC(18:2/0:0)	18.83	520.3424	M^+^	C_26_H_51_NO_7_P^+^	520.67	520.3409	2.95	184.0744, 123.0812, 86.0968	-	-	-	t
23	5-hydroxy-1-(4-hydroxy-3-methoxyphenyl)decan-3-one	16.44/15.32	295.1891/295.1916	[M + H]^+^	C_17_H_26_O_4_	294.39	295.1915	−8.07/0.40	239.1278, 221.1161, 193.1218, 139.1109, 123.0797, 101.0229, 85.0279	-	-	t	t
24	SN-Glycero-3-Phosphocholine	0.95/0.92/0.53	258.1090/258.1089/258.1114	[M + H]^+^	C_8_H_20_NO_6_P	257.22	258.1112	−8.51/−8.90/0.79	258.1088, 184.0724, 124.9991, 104.1064, 86.0958	-	t	t	t
25	Aurofusarin	17.57/17.68/14.59	571.0853/571.0852/571.0903	[M + H]^+^	C_30_H_18_O_12_	570.46	571.0882	−5.08/−5.25/3.68	556.0668, 541.0425, 528.0727, 511.0689, 484.0820	t	t	-	t
26	Fusarin C	13.88	454.1854	[M + Na]^+^	C_23_H_29_NO_7_	431.49	454.1847	1.49	426.1905, 335.1276, 290.1012, 267.1372, 250.0722, 222.0664, 69.4139	t	-	t	+
27	Lucilactaene	16.68/14.85	438.1874/438.1908	[M + Na]^+^	C_23_H_29_NO_6_	415.49	438.1898	−5.49/2.27	274.1065	-	-	t	+
28	Fusaristatin A	20.67/20.66/20.65	659.4354/659.4355/659.4357	[M + H]^+^	C_36_H_58_N_4_O_7_	658.43	659.4389	−5.34/−5.19/−4.89	428.3155, 377.3022, 359.2934, 331.2983, 303.2669, 232.1282	+	+	+	-
29	(E)-3-(6,13-dimethyl-10-methylene-2,5,9,12-tetraoxo-14-(3,7,11-trimethyl-4-oxoheptadec-7-en-1-yl)-1-oxa-4,8,11-triazacyclotetradecan-3-yl)propanamide	20.73/20.72/20.72/19.85	661.4513/661.4512/661.4511/661.4564	[M + H]^+^	C_36_H_60_N_4_O_7_	660.45	661.4545	−4.95/−5.10/−5.25/2.76	430.3304, 402.3347, 359.2926, 331.2975, 303.2666, 232.1280	+	+	+	+
30	(E)-3-(13-methyl-10-methylene-2,5,9,12-tetraoxo-14-(3,7,11-trimethyl-4-oxoheptadec-7-en-1-yl)-1-oxa-4,8,11-triazacyclotetradecan-3-yl)propanamide	20.70/20.68/20.68/19.86	647.4356/647.4357/647.4356/647.4410	[M + H]^+^	C_35_H_58_N_4_O_7_	646.87	647.4389	−5.13/−4.98/ −5.13/3.21	430.3298, 402.3349, 359.2927, 303.2666, 218.1126, 147.0756	t	t	t	+
31	(Z)-3-(hydroxy(6-hydroxy-1-methyl-2-(2-methylprop-1-en-1-yl)-1,2-dihydronaphthalen-1-yl)methylene)-5-(hydroxymethyl)-1-methylpyrrolidine-2,4-dione	14.82/14,79/14.79/14.84	384.1791/384.1791/384.1793/384.1825	[M + H]^+^	C_22_H_25_NO_5_	383.44	384.1816	−6.63/−6.63/−6.11/2.22	384.1824, 366.1726, 338.1772, 241.1237, 213.1287	t	t	t	t

^a^ *Fusarium* sp. VM-40 control group; ^b^ *Fusarium* sp. VM-40 grown on media with 1 mM sodium butyrate; ^c^ *Fusarium* sp. VM-40 grown on media with 10 mM sodium butyrate; ^d^ *Fusarium* sp. VM-40 grown on media with 100 mM sodium butyrate. “-”, not detected; “t”, trace amounts were detected; “+”, detectable. Similarly, compound **31** in cluster 5 is predicted to be (Z)-3-(hydroxy(6-hydroxy-1-methyl-2-(2-methylprop-1-en-1-yl)-1,2-dihydronaphthalen-1-yl)methylene)-5-(hydroxymethyl)-1-methylpyrrolidine-2,4-dione, an equisetin derivative that is likely associated with the equisetin BGC (region 19.1). In our experiments, we observed that only 100 mM SB could trigger this BGC and elicit the production of equisetin analogs.

## Data Availability

The sequencing data and genome assembly for this study have been deposited in the European Nucleotide Archive (ENA) at EMBL-EBI under the accession number PRJEB62500. The mass spectrometry data have been deposited on GNPS under the accession number MassIVE ID: MSV000092159.

## Supplementary Materials

The following supporting information can be downloaded at: https://www.mdpi.com/article/10.3390/jof9070704/s1. Supplementary Information file with Figure S1. Contigs of *Fusarium* sp. VM-40 visualized by Bandge; Figure S2. Taxonomic tree generated by comparing 3613 single copy orthologous genes from the genomes of 22 *Fusarium* species using OrthoFinder; Figure S3. Maximum-Likelihood (ML) phylogram of FTSC species based on the *tef1* gene; Figure S4. Chemical structures of the secondary metabolites predicted by antiSMASH; Figure S5. Proposed fragmentations of compound **8**; Figure S6. Proposed fragmentations of compound **10**; Table S1. List of *Fusarium* species from JGI used for comparative analysis and phylogenetic analysis; Table S2. List of *Fusarium* isolates belonging to the *F. tricinctum* species complex (FTSC) from NCBI; Table S3. Quality metrics for isolated genomic DNA; Table S4. The QUAST statistics of *Fusarium* sp. VM-40; Table S5. Gene distribution of *Fusarium* sp. VM-40 based on the six major modules of CAZymes; Table S6. Biosynthetic gene clusters of *Fusarium* sp. VM-40 predicted by antiSMASH; Dataset S1. Matches of ITS sequencing blasted against the NCBI nr/nt database; Dataset S2. Overall statistics of single-copy orthologous genes from the genomes of 22 *Fusarium* species using OrthoFinder; Dataset S3. Molecular network of SMs of *Fusarium* sp. VM-40 in Cytoscape. References [69,70,71,72,73,74,75,76,77,78,79,80,81] are cited in the Supplementary Materials.

## Abbreviations

AAs: auxiliary activities; BGCs: biosynthetic gene clusters; BLAST: Basic Local Alignment Search Tool; CAZymes: carbohydrate-active enzymes; CBMs: carbohydrate-binding modules; CYA: Czapek yeast autolysate agar; CEs: carbohydrate esterases; ENNs: enniatins; FTSC: *F*. *tricinctum* species complex; GenSAS: genome sequence annotation server; GHs: glycoside hydrolases; GNPS: Global Natural Products Social Molecular Networking; GO: Gene Ontology; GTs: glycosyl transferases; HAS: hexadehydroastechrome; Hiv: 2-hydroxy isovaleric acid; HPLC: high-performance liquid chromatography; HR-LC-MS: high-resolution liquid chromatography-mass spectrometry; ITS: Internal transcribed spacer; LSU: large subunit ribosomal RNA; ML: Maximum-Likelihood; NCBI: National Center for Biotechnology Information; NMeAla: N-methyl-alanine; NMeIle: N-methyl-isoleucine; NMeLeu: N-methyl-leucine; NMeThr: N-methyl-threonine; NMeVal: aliphatic N-methyl-valine; NRPS: nonribosomal peptide synthetase; PCR: polymerase chain reaction; PDA: potato dextrose agar; PKS: polyketide synthase; PLs: polysaccharide lyases; rt: retention time; RPB1: RNA polymerase II subunit I; RPB2: RNA polymerase II second largest subunit; rRNA: ribosomal RNA; SB: sodium butyrate; SMs: secondary metabolites; SNA: synthetically nutrient-poor agar; TEF1: translation elongation factor 1 α; tRNA: transfer RNA; TUB2: beta-tubulin.
