# Supplementary material for: Genomic and Metabolomic Analysis of the Endophytic Fungus Fusarium sp. VM-40 Isolated from the Medicinal Plant Vinca minor
Source: J Fungi (Basel). 2023 Jun 27;9(7):704. doi: 10.3390/jof9070704 (PMC10381429; doi:10.3390/jof9070704)
Supplement: Supplementary file 1 [file jof-09-00704-s001.zip › Supplementary file.pdf]

Genomic and metabolomic analysis of the endophytic fungus *Fusarium sp.* VM-40 isolated from the medicinal plant *Vinca minor*

Ting He[1], Xiao Li[1], Riccardo Iacovelli [1], Thomas Hackl[2] and Kristina Haslinger[1] *

[1] Department of Chemical and Pharmaceutical Biology, Groningen Research Institute of Pharmacy, University of Groningen, Antonius Deusinglaan 1, 9713 AV Groningen, The Netherlands

[2] Groningen Institute of Evolutionary Life Sciences, University of Groningen, Nijenborgh 7, 9747 AG Groningen, The Netherlands

Table S1. List of *Fusarium* species from JGI used for comparative analysis and phylogenetic analysis.

Table S2. List of *Fusarium* isolates belonging to the *F. tricinctum* species complex (FTSC) from NCBI.

Table S3. Quality metrics for isolated genomic DNA.

Table S4. The QUAST statistics of *Fusarium sp*. VM-40.

Table S5. Gene distribution of *Fusarium sp.* VM-40 based on the six major modules of CAZymes.

Table S6. Biosynthetic gene clusters of *Fusarium sp*. VM-40 predicted by antiSMASH.

Figure S1. Contigs of *Fusarium sp*. VM-40 visualized by Bandge.

Figure S2. Taxonomic tree generated by comparing 3613 single copy orthologous genes from the genomes of 22 *Fusarium* species using OrthoFinder

Figure S3. Maximum-Likelihoood (ML) phylogram of FTSC species based on the comparison of the *tef1* gene

Figure S4. Chemical structures of the secondary metabolites predicted by antiSMASH.

Figure S5. Proposed fragmentations of compound **8**.

Figure S6. Proposed fragmentations of compound **10**.

Table S1. List of *Fusarium* species from JGI used for comparative analysis and phylogenetic analysis.

Species	Strain	Reference
Fusarium avenaceum	MPI-SDFR-AT-0044	[1]
Fusarium bataticola	FSSC 23	[2]
Fusarium commune	MPI-SDFR-AT-0072	[1]
Fusarium cucurbiticola	FSSC 10	[2]
Fusarium culmorum	UK99	[3]
Fusarium fujikuroi	IMI 58289	[4]
Fusarium graminearum	Z3639	[2]
Fusarium oxysporum	Fo47	[5]
Fusarium poae	2516	[6]
Fusarium proliferatum	ET1	[7]
Fusarium pseudograminearum	CS3096	[8]
Fusarium redolens	MPI-CAGE-AT-0023	[1]
Fusarium solani f. mor	NRRL 22230	[9]
Fusarium tenuicristatum	NRRL 22470	[10-11]
Fusarium tricinctum	F194	[1]
Fusarium vanettenii	T33	[9]
Fusarium venenatum	MPI-CAGE-CH-0201	[1]
Fusarium verticillioides	7600	[12]
Neonectria ditissima	R0905	[13]

Table S2. List of *Fusarium* isolates belonging to the *F. tricinctum* species complex (FTSC) from NCBI.

Species	Strain	TEF1
Fusarium alpinum	LC6043	MW620149.1
Fusarium alpinum	LC6034	MW620147.1
Fusarium avenaceum	LC6387	MW620132.1
Fusarium avenaceum	LC13803	MW620118.1
Fusarium acuminatum	NRRL 36147	OL772880.1
Fusarium arthrosporioides	BCCM/IHEM 14084	KJ126123.1
Fusarium paeoniae	LC5166	MW620143.1
Fusarium paeoniae	LC7358	MW620144.1
Fusarium paeoniae	LC13815	MW620140.1
Fusarium chongqingense	LC13813	MW620136.1
Fusarium tricinctum	CBS 253.50	KR071775.1
Fusarium tricinctum	NRRL 36381	OL772830.1
Fusarium sp. FTSC_5	NRRL 52727	OL772885.1
Fusarium sp. FTSC_5	NRRL 52726	OL772884.1
Fusarium sp. FTSC_8	NRRL 52722	OL772890.1
Fusarium sp. FTSC_11	P2289a	OL964855.1
Fusarium sp. FTSC_12	P2285a	OL964770.1
Fusarium sp. FTSC_13	F164	OL964757.1
Fusarium sp. FTSC_14	R972	OL964801.1
Fusarium sp. FTSC_15	P78c	OL964788.1
Fusarium sp. FTSC_17	NRRL 53952	OL772853.1
Fusarium sp. FTSC_18	NRRL 36565	OL772876.1
Fusarium sp. FTSC_19	FRC R3178	OL772777.1
Fusarium sp. FTSC_19	FRC R3120	OL772776.1
Fusarium sp. FTSC_10	NRRL 52720	OL772893.1
Fusarium sp. FTSC_20	FRC R7848	OL772774.1
Fusarium sp. FTSC_21	NRRL 39843	OL772788.1
Fusarium sp. FTSC_22	FRC R6739	OL772764.1
Fusarium sp. FTSC_23	FRC R7739	OL772835.1
Fusarium sp. FTSC_24	FRC R7356	OL772894.1
Fusarium sp. FTSC_25	FRC R4587	OL772866.1
Fusarium iranicum	NRRL 54147	OL772891.1
Fusarium flocciferum	NRRL 45999	OL772882.1
Fusarium petersiae	CBS 143231	MG386159.1
Fusarium torulosum	NRRL 22748	OL772877.1
Neonectria ditissima	R0905	-

Table S3. Quality metrics for isolated genomic DNA.

Qubit conc. (ng/µl)	95.0
NanoDrop conc. (ng/µl)	123.9
Output (µg)	5.7
A260/280 ratio	1.80
A260/230 ratio	2.04
NanoDrop/Qubit conc. ratio	1.3
Qubit conc. after Circulomics (ng/µl)	83.4
Input for library preparation (µg)	1
Output after DNA repair and end-prep (µg)	0.978
Output after adapter ligation and clean-up (µg)	0.756

Table S4. QUAST statistics of the *Fusarium sp*. VM-40 whole genome assembly.

Statistics without reference	assembly
# contigs (>= 0 bp)	19
# contigs (>= 1000 bp)	19
# contigs (>= 5000 bp)	18
# contigs (>= 10000 bp)	18
# contigs (>= 25000 bp)	16
# contigs (>= 50000 bp)	15
Total length (>= 0 bp)	42057343
Total length (>= 1000 bp)	42057343
Total length (>= 5000 bp)	42054254
Total length (>= 10000 bp)	42054254
Total length (>= 25000 bp)	42021767
Total length (>= 50000 bp)	41979816
# contigs	19
Largest contig	5654227
Total length	42057343
GC (%)	47.72
N50	4299955
N90	1527832
L50	5
L90	11

Table S5. Gene distribution of *Fusarium sp.* VM-40 based on the six major modules of CAZymes.

**Module family**	**Module**	**Numbers**
Glycoside hydrolases (GH)	GH1	2
	GH10	2
	GH105	4
	GH106	1
	GH109	2
	GH11	1
	GH114	3
	GH115	2
	GH12	3
	GH125	2
	GH13	4
	GH131	1
	GH132	1
	GH135	1
	GH142	2
	GH15	1
	GH152	1
	GH154	2
	GH16	15
	GH17	5
	GH18	11
	GH2	10
	GH20	3
	GH27	2
	GH28	8
	GH29	1
	GH3	29
	GH31	8
	GH32	6
	GH33	1
	GH35	4
	GH36	4
	GH37	2
	GH38	1
	GH39	2
	GH43	27
	GH45	1

	GH47	9
	GH49	1
	GH5	23
	GH51	2
	GH53	1
	GH54	1
	GH55	3
	GH6	1
	GH62	2
	GH63	1
	GH64	1
	GH65	1
	GH67	1
	GH7	1
	GH71	1
	GH72	2
	GH74	1
	GH75	3
	GH76	7
	GH78	7
	GH81	1
	GH88	3
	GH92	1
	GH93	5
	GH95	1
Auxiliary activities (AAs)	AA1	11
	AA11	4
	AA12	2
	AA13	1
	AA16	1
	AA2	5
	AA3	25
	AA4	3
	AA5	5
	AA6	1
	AA7	22
	AA8	4
	AA9	12
Glycosyltransferases (GTs)	GT1	16
	GT109	3

	GT15	4
	GT17	1
	GT2	17
	GT20	3
	GT21	1
	GT22	4
	GT24	1
	GT3	1
	GT31	5
	GT32	6
	GT33	1
	GT34	3
	GT35	1
	GT39	3
	GT4	6
	GT41	1
	GT48	1
	GT50	1
	GT57	2
	GT58	1
	GT59	1
	GT62	3
	GT64	2
	GT66	1
	GT69	3
	GT71	3
	GT76	1
	GT8	6
	GT90	6
Carbohydrate esterases (CEs)	CE1	4
	CE12	3
	CE16	6
	CE18	1
	CE2	1
	CE3	10
	CE4	9
	CE5	11
	CE8	3
	CE9	1
Carbohydrate-binding modules	CBM1	2

(CBMs)		
	CBM13	1
	CBM18	14
	CBM20	1
	CBM21	1
	CBM24	2
	CBM35	3
	CBM42	2
	CBM43	2
	CBM48	2
	CBM50	8
	CBM6	1
	CBM63	3
	CBM67	4
	CBM87	1
	CBM91	7
Polysaccharide lyases (PLs)	PL1	11
	PL11	1
	PL26	1
	PL3	9
	PL4	3
	PL42	2
	PL9	2

Table S6. Biosynthetic gene clusters of *Fusarium sp*. VM-40 predicted by antiSMASH.

**Cluster ID**	**Region**	**Contig**	**Type**	**From**	**To**	**Most similar known cluster**	**Genbank**	**Type**	**Similarity**
1	1.1	14	NRPS-like	295355	353717				
2	1.2	14	Terpene	763502	794776	α-acorenol	HF679032.1	Terpene	1
3	1.3	14	NRPS	1205989	1272242				
4	2.1	5	NRPS, NRPS-like, T1PKS	164599	297127	Fusatrixin/fusapentaxin/fusaoctaxin A	CM000576.1	NRP	0.87
5	2.2	5	NRPS, T1PKS	415050	486937	ACT-Toxin II	AB516321.1	Polyketide	1
	2.3	5	Fungal-RiPP-like	614114	705241				
6	3.1	21	T3PKS, NRPS	43028	157388	Peramine/intermediate 1/intermediate 2	MH938357.1	NRP	1
7	3.2	21	NRPS	216154	289015				
9	3.3	21	Indole	761991	793241				
10	3.4	21	Terpene, NRPS	1278270	1407628	Culmorin/(+)-juniperol(longiborneol)/ 15-acetyldeoxynivalenol	CM000574.1	Terpene	1
11	3.5	21	Fungal-RiPP-like	1600921	1694603				
12	3.6	21	T1PKS	1697028	1765648	Gibepyrone-A	HF679033.1	Polyketide	0.4
13	5.1	24	T1PKS	761308	828456	Oxyjavanicin	HE613440.1	Polyketide	0.87
14	5.2	24	Terpene	1399539	1432090	Squalestatin S1	LDZW01000177.1	Terpene	0.4

15	5.3	24	NRP-metallophore, NRPS	2598020	2681533				
16	6.1	18	Terpene	35601	68710	Gibberellin	KT216505.1	Terpene	0.85
17	6.2	18	NRPS	149800	232668				
18	7.1	15	T1PKS	13788	82708				
19	7.2	15	Fungal-RiPP-like	233298	324179				
20	7.3	15	Terpene	418888	450465				
21	7.4	15	T1PKS	495048	576701	Lijiquinone	KT874412.1	Polyketide	0.62
22	8.1	22	T1PKS	306667	371773				
23	8.2	22	Phosphonate	407924	434676	Fosfonochlorin	CM000596.1	Other	0.76
24	8.3	22	T1PKS, NRPS	438153	567620	Ilicicolin H/ilicicolin J/8-epi-ilicicolin H	RQWH01000002.1	NRP + Polyketide	0.83
25	8.4	22	NRPS	899797	963512				
26	8.5	22	T3PKS	1080248	1141718				
27	8.6	22	T1PKS	2405290	2472764				
28	9.1	9	NRPS	983271	1052669	Beauvericin	EU886196.1	NRP	0.2
29	10.1	4	NRPS-like	1307934	1368649	Bassianolide	FJ439897.1	NRP	0.26
30	10.2	4	Terpene	2028925	2060184				
31	10.3	4	NRPS	2106488	2170172	Chrysogine	NC_026476.1	NRP	1

32	10.4	4	T1PKS, NRPS	2220936	2375153	Lucilactaene	LC515193.1	Polyketide	0.61
33	11.1	27	T1PKS	1	62583				
34	11.2	27	Fungal-RiPP-like	450256	541207				
35	13.1	11	T1PKS	54592	124703				
36	13.2	11	NRPS	452458	520206				
37	13.3	11	Terpene	1442368	1473495				
38	14.1	19	NRPS, Indole	32215	97591	Hexadehydroastechrome/terezine-D/astechrome	CM000171.1	NRP	1
39	14.2	19	T1PKS	170110	230638	Aurofusarin	CM000574.1	Polyketide	0.9
40	14.3	19	NRPS, T1PKS	289487	372153	Fusaristatin A	CM000575.1	NRP + Polyketide	1
41	14.4	19	Terpene	2852862	2883988				
42	15.1	3	T1PKS	605930	683015	Orcinol/orsellinic acid	CM000575.1	Polyketide	0.55
43	15.2	3	Fungal-RiPP-like	2245197	2338262				
44	15.3	3	NRPS-like	4077446	4140787				
45	15.4	3	Indole	4203804	4235078				
46	17.1	7	T1PKS, T3PKS	28890	94430				
47	17.2	7	Terpene	158798	189551	PR-toxin	HG792016.1	Terpene	0.5
48	17.3	7	NRPS, T1PKS	390515	462676	Fusaridione A/ (3R,5S)-5-(4-	AY700570.3	NRP + Polyketide	0.18

						hydroxybenzyl)-3-methyl-3-((2E,4E,6E,8E,10E)-4,8,10-trimethyldodeca-2,4,6,8,10-pentaenoyl)pyrrolidine-2,4-dione			
49	17.4	7	NRPS-like	646679	710753				
50	17.5	7	NRPS-like	794850	858701	Choline	CH236925.1	NRP	1
51	17.6	7	Betalactone	1207360	1249717				
52	19.1	8	NRPS-like	447125	509494	Equisetin	KC439347.1	NRP + Polyketide	0.18
53	19.2	8	NRPS	514479	591098				
54	19.3	8	Fungal-RiPP-like	612755	703644				
55	19.4	8	Terpene	823883	853011	Koraiol	HF679030.1	Terpene	1
56	19.5	8	Terpene	1209165	124177				

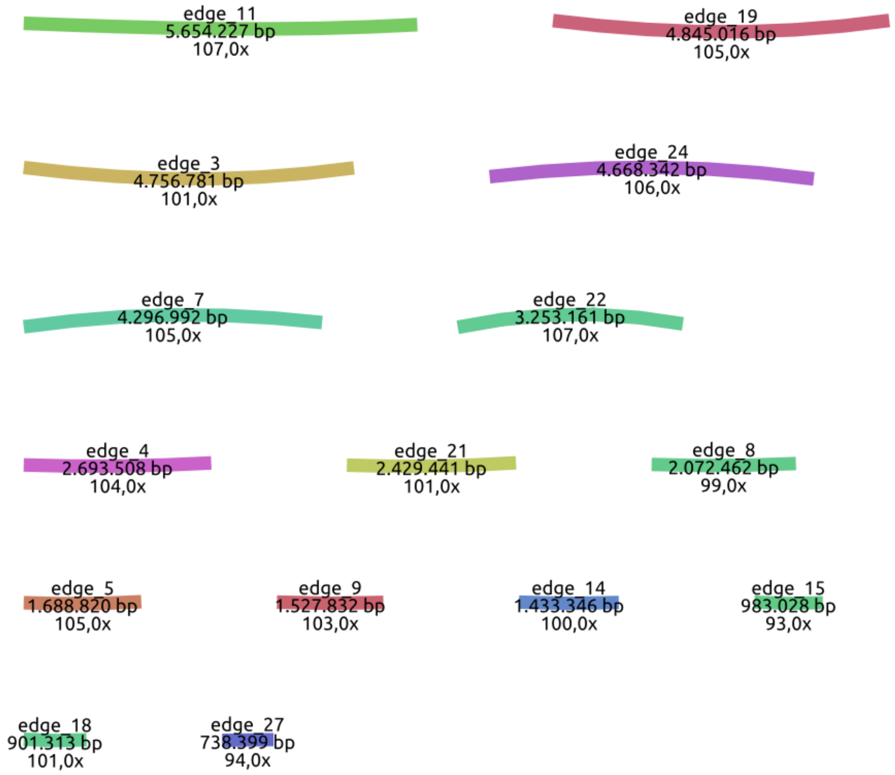

Figure S1. Contigs of *Fusarium sp*. VM-40 visualized by Bandage.

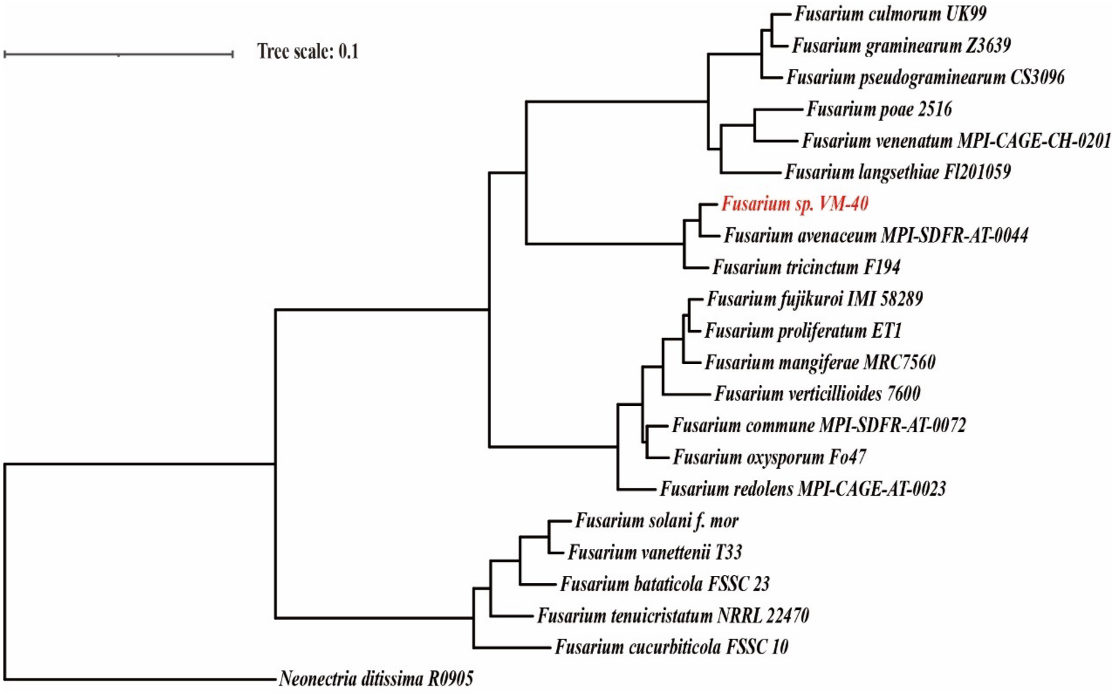

Figure S2. Taxonomic tree generated by comparing 3613 single copy orthologous genes from the genomes of 22 *Fusarium* species using OrthoFinder. Phylogenetic tree was conducted using FastTree with local bootstrap values of 1000 replicates.

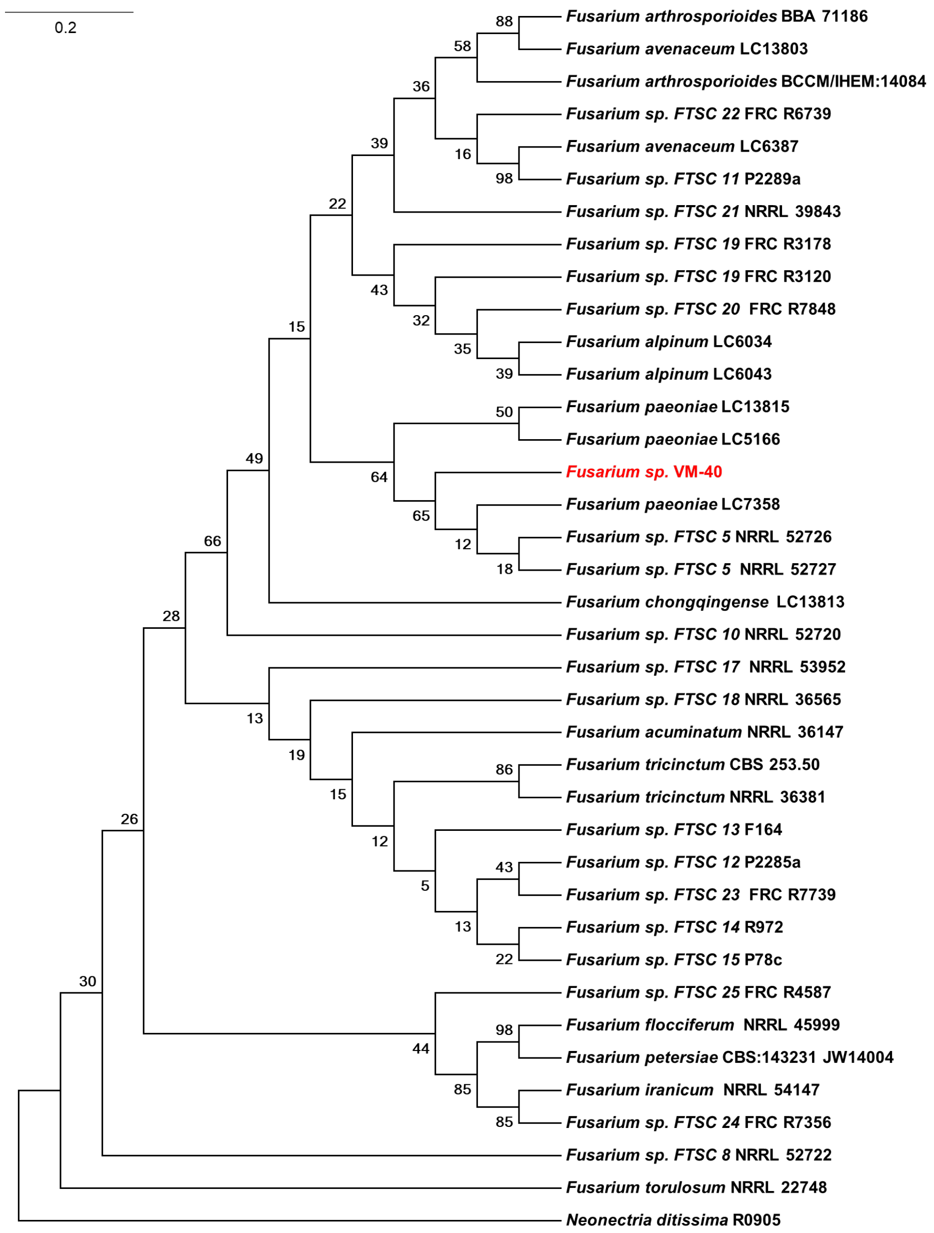

Figure S3. Maximum-Likelihoood (ML) phylogram of FTSC species based on the comparison of the *tef1* gene. Phylogenetic tree was conducted using IQ-Tree with local bootstrap values of 1000 replicates.

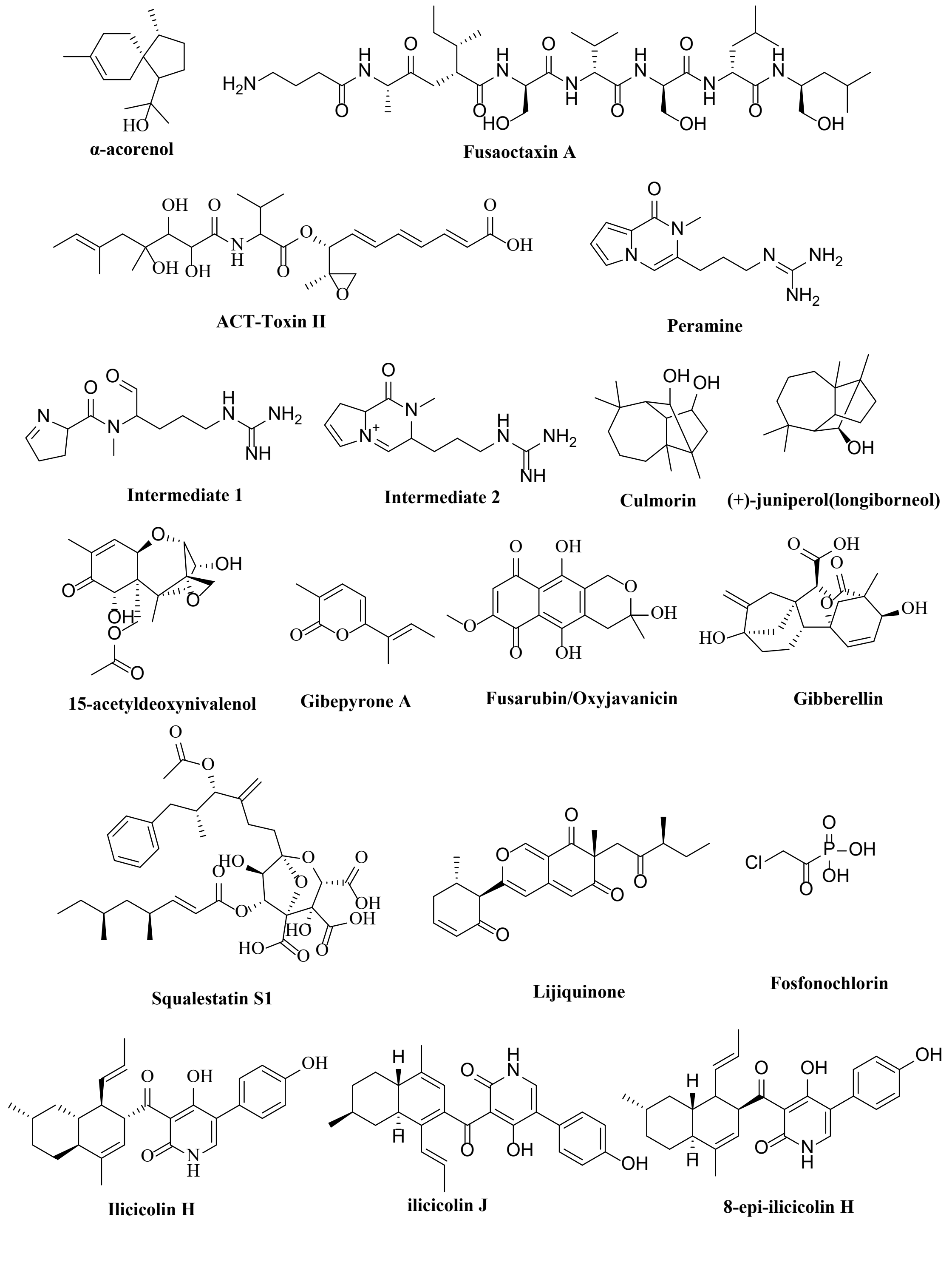
α-acorenol
Fusaoctaxin A
ACT-Toxin II
Peramine
Intermediate 1
Intermediate 2
Culmorin
(+)-juniperol(longiborneol)
15-acetyldeoxynivalenol
Gibepyrone A
Fusarubin/Oxyjavanicin
Gibberellin
Squalestatin S1
Lijiquinone
Fosfonochlorin
Ilicicolin H
ilicicolin J
8-epi-ilicicolin H

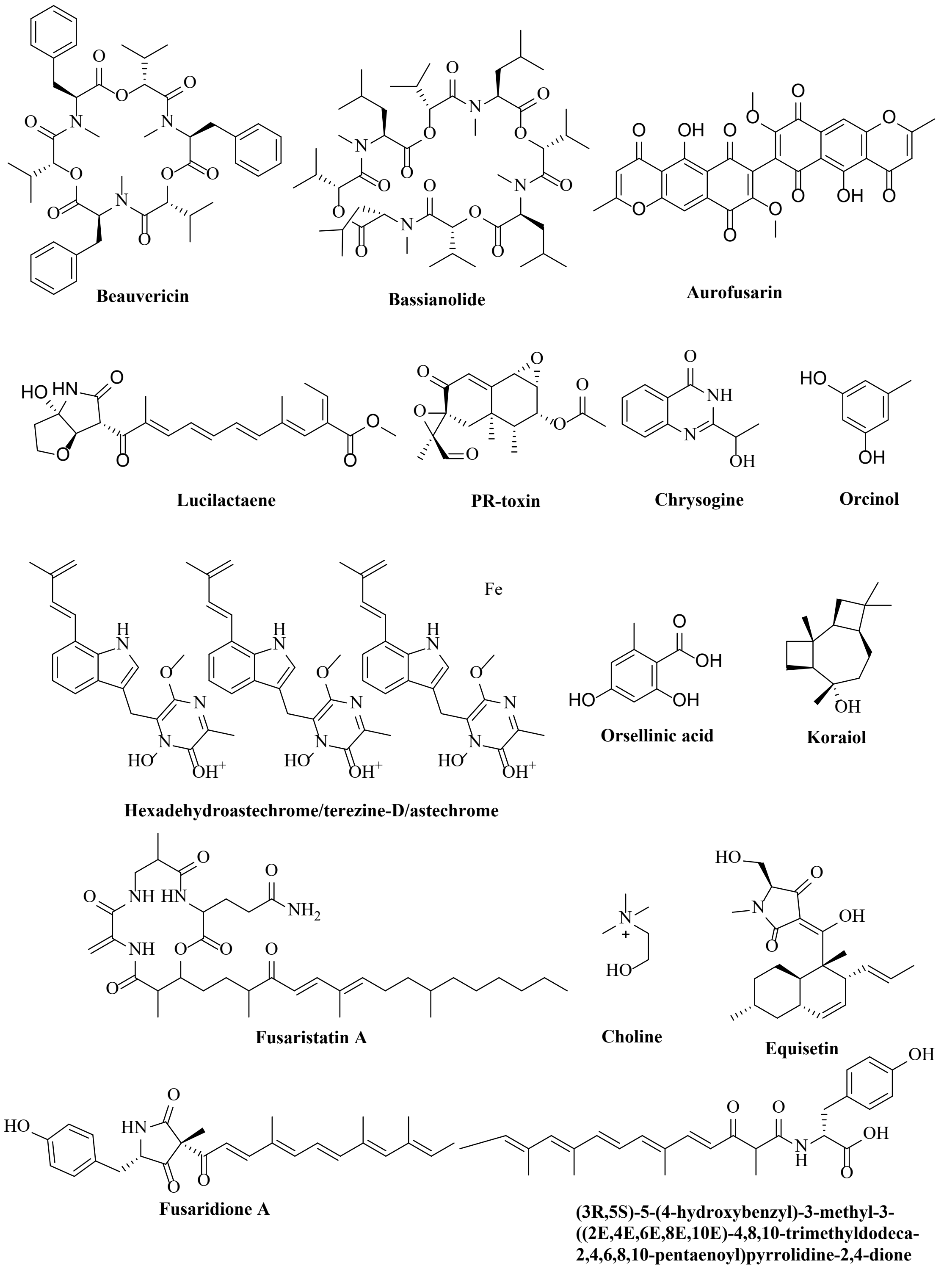

Figure S4. Chemical structures of the types of secondary metabolites that *Fusarium* sp. VM-40 may be able to produce based on the presence of known BGCs in its genome predicted by antiSMASH.

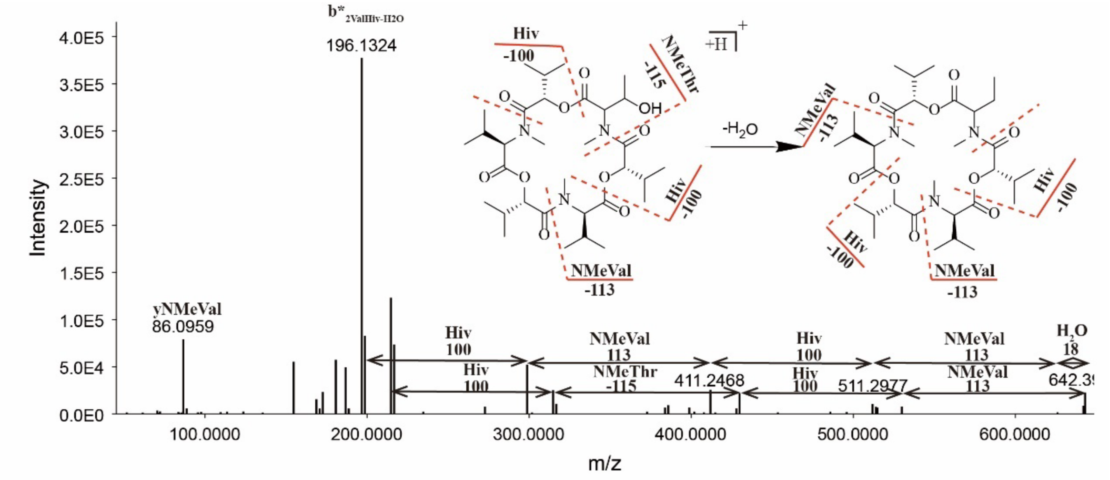

Figure S5. Proposed fragmentations of compound **8:** $[M+H]^+$ - *m*/*z* 642.3979, $[M+H\text{-methyl-valine (NMeVal)}]^+$ - *m*/*z* 529.3160, $[M+H\text{-NMeVal-2-hydroxy-isovaleric acid (Hiv)}]^+$ - *m*/*z* 429.2618, $[M+H\text{-NMeVal-Hiv-N-methyl-Threonine(NMeThr)}]^+$ - *m*/*z* 314.1987, $[M+H\text{-NMeVal-Hiv-NMeThr-Hiv}]^+$ - *m*/*z* 214.1449, [M+H-H_2O] - *m*/*z* 624.3943, [M+H-H_2O-NMeVal] - *m*/*z* 511.3055, [M+H-H_2O-NMeVal-Hiv] - *m*/*z* 411.2516, [M+H-H_2O-NMeVal-Hiv-NMeVal] - *m*/*z* 298.1658, [M+H-H_2O-NMeVal-Hiv-NMeVal-Hiv] - *m*/*z* 196.1343, y_{NMeVal} - *m*/*z* 86.0968, $b^*_{2ValHiv}$-H_2O - *m*/*z* 196.1343

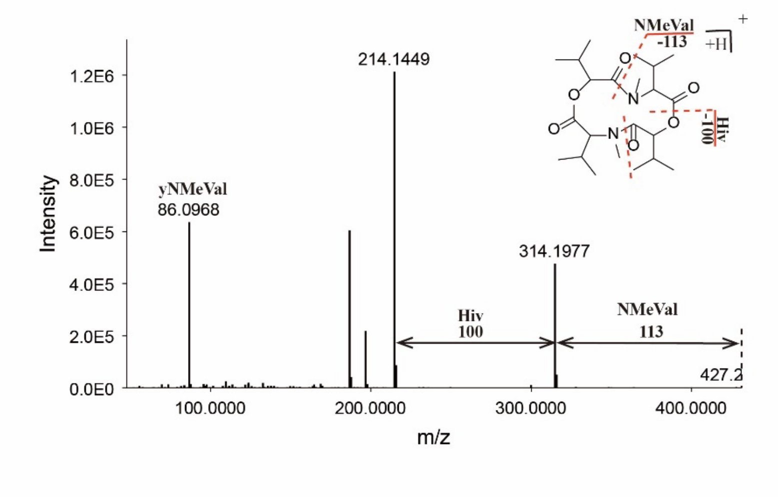

Figure S6. Proposed fragmentations of compound **10** (m/z 427.282).

Reference

1. Mesny, F.; Miyauchi, S.; Thiergart, T.; Pickel B.; Atanasova, L.; Karlsson, M.; Hüttel, B.; Barry, K.W.; Haridas, S.; Chen, C.; Bauer, D.; Andreopoulos, W.; Pangilinan J.; LaButti, K.; Riley, R.; Lipzen, A.; Clum, A.; Drula, E.; Henrissat, B.; Kohler, A.; Grigoriev, I.V.; Martin, F.M.; Hacquard, S. Genetic determinants of endophytism in the *Arabidopsis* root mycobiome. *Nat. Commun*. **2021**, *12*, 7227.
2. Nordberg, H.; Cantor, M.; Dusheyko, S.; Hua, S.; Poliakov, A.; Shabalov, I.; Smirnova, T.; Grigoriev, I.V.; Dubchak, I. The genome portal of the Department of Energy Joint Genome Institute: 2014 updates. *Nucleic Acids Res.* **2014**, *42*, 26-31.
3. Urban, M.; King, R.; Andongabo, A.; Maheswari, U.; Pedro, H.; Kersey, P.; Hammond-Kosack, K. First draft genome sequence of a UK strain (UK99) of *Fusarium culmorum*. *Genome Announc.* **2016**, *4*, e00771-16.
4. Wiemann, P.; Sieber, C.M.; von Bargen, K.W.; Studt, L.; Niehaus, E.M.; Espino, J.J.; Huß, K.; Michielse, C.B.; Albermann, S.; Wagner, D.; Bergner, S.V.; Connolly, L.R.; Fischer, A.; Reuter, G.; Kleigrewe, K.; Bald, T.; Wingfield, B.D.; Ophir, R.; Freeman, S.; Hippler, M.; Smith, KM.; Brown, D.W.; Proctor, R.H.; Münsterkötter, M.; Freitag, M.; Humpf, H.U.; Güldener, U.; Tudzynski, B. Deciphering the cryptic genome: genome-wide analyses of the rice pathogen *Fusarium fujikuroi* reveal complex regulation of secondary metabolism and novel metabolites. *PLoS Pathog.* **2013**, *9*, e1003475.
5. Wang, B.; Yu, H.; Jia, Y.; Dong, Q.; Steinberg, C.; Alabouvette, C.; Edel-Hermann, V.; Kistler, H.C.; Ye, K.; Ma, L.J.; Guo, L. Chromosome-scale genome assembly of *Fusarium oxysporum* strain Fo47, a fungal endophyte and biocontrol agent. *Mol. Plant Microbe. Interact.* **2020**, *33*, 1108-1111.
6. Vanheule, A.; Audenaert, K.; Warris, S.; van, de, Geest, H.; Schijlen, E.; Höfte, M.; De, Saeger, S.; Haesaert, G.; Waalwijk, C.; van, der, Lee, T. Living apart together: crosstalk between the core and supernumerary genomes in a fungal plant pathogen. *BMC Genom.* **2016**, *23*, 670.
7. Niehaus, E.M.; Münsterkötter, M.; Proctor, R.H.; Brown, D.W.; Sharon, A.; Idan, Y.; Oren-Young, L.; Sieber, C.M.; Novák, O.; Pěnčík, A.; Tarkowská, D.; Hromadová, K.; Freeman, S.; Maymon, M.; Elazar, M.; Youssef, S.A.; El-Shabrawy, E.S.; Shalaby, A.B.; Houterman, P.; Brock, N.L.; Burkhardt, I.; Tsavkelova, E.A.; Dickschat, J.S.; Galuszka, P.; Güldener, U.; Tudzynski, B. Comparative "Omics" of the *Fusarium fujikuroi* species complex highlights differences in genetic potential and metabolite Synthesis. *Genome Biol. Evol.* **2016**, *31*, 3574-3599.
8. Gardiner, D.M.; McDonald, M.C.; Covarelli, L.; Solomon, P.S.; Rusu, A.G.; Marshall, M.; Kazan, K.; Chakraborty, S.; McDonald, B.A.; Manners, J.M. Comparative pathogenomics reveals horizontally acquired novel virulence genes in fungi infecting cereal hosts. *PLoS Pathog.* **2012**, *8*, e1002952.
9. Coleman, J.J.; Rounsley, S.D.; Rodriguez-Carres, M.; Kuo, A.; Wasmann, C.C.; Grimwood, J.; Schmutz, J.; Taga, M.; White, G.J.; Zhou, S.; Schwartz, D.C.; Freitag, M.; Ma, L.J.; Danchin, E.G.; Henrissat, B.; Coutinho, P.M.; Nelson, D.R.; Straney, D.; Napoli, C.A.; Barker, B.M.; Gribskov, M.; Rep, M.; Kroken, S.; Molnár, I.; Rensing, C.; Kennell, J.C.; Zamora, J.; Farman, M.L.; Selker, E.U.; Salamov, A.; Shapiro, H.; Pangilinan, J.; Lindquist, E.; Lamers, C.; Grigoriev, I.V.; Geiser, D.M.; Covert, S.F.; Temporini, E.; Vanetten, H.D. The genome of *Nectria haematococca*: contribution of supernumerary chromosomes to gene expansion. *PLoS Genet.* **2009**, *5*, e1000618.
10. Temporini, E.D. and VanEtten, H.D. An analysis of the phylogenetic distribution of the pea pathogenicity genes in *Nectria haematococca* MPVI supports the hypothesis of their origin by

horizontal transfer and uncovers a potentially new pathogen of garden pea: *Neocosmospora boniensis*. *Curr. Genet.* **2004**, *46*, 29-36.

11. Udagawa, S.I..; Horie, Y..; Cannon, P.F. Two new species of *Neocosmospora* from Japan, with a key to the currently accepted species. *Sydowia* **1989**, *41*: 349-359.
12. Ma, L.J.; van, der, Does, H.C.; Borkovich, K.A.; Coleman, J.J.; Daboussi, M.J.; Di, Pietro, A.; Dufresne, M.; Freitag, M.; Grabherr, M.; Henrissat, B.; Houterman, P.M.; Kang, S.; Shim, W.B.; Woloshuk, C.; Xie, X.; Xu, J.R.; Antoniw, J.; Baker, S.E.; Bluhm, B.H.; Breakspear, A.; Brown, D.W.; Butchko, R.A.; Chapman, S.; Coulson, R.; Coutinho, P.M.; Danchin, E.G.; Diener, A.; Gale, L.R.; Gardiner, D.M.; Goff, S.; Hammond-Kosack, K.E.; Hilburn, K.; Hua-Van, A.; Jonkers, W.; Kazan, K.; Kodira, C.D.; Koehrsen, M.; Kumar, L.; Lee, YH.; Li, L.; Manners, J.M.; Miranda-Saavedra, D.; Mukherjee, M.; Park, G.; Park, J.; Park, S.Y.; Proctor, R,H.; Regev, A.; Ruiz-Roldan, M.C.; Sain, D.; Sakthikumar, S.; Sykes, S.; Schwartz, D.C.; Turgeon, B.G.; Wapinski, I.; Yoder, O.; Young, S.; Zeng, Q.; Zhou, S.; Galagan, J.; Cuomo, C.A.; Kistler, H.C.; Rep, M. Comparative genomics reveals mobile pathogenicity chromosomes in *Fusarium*. *Nature* **2010**, *464*, 367-73.
13. Gómez-Cortecero, A.; Harrison, R.J.; Armitage, A.D. Draft genome sequence of a European isolate of the apple canker pathogen *Neonectria ditissima*. *Genome Announc.* **2015**, *3*, e01243-15.
